# Evaluation of the neuroprotective efficacy of the gramine derivative ITH12657 against NMDA-induced excitotoxicity in the rat retina

**DOI:** 10.3389/fnana.2024.1335176

**Published:** 2024-02-13

**Authors:** Johnny Di Pierdomenico, Alejandro Gallego-Ortega, María Norte-Muñoz, Beatriz Vidal-Villegas, Isaac Bravo, María Boluda-Ruiz, Jose Manuel Bernal-Garro, Iván Fernandez-Bueno, Jose Carlos Pastor-Jimeno, María Paz Villegas-Pérez, Marcelino Avilés-Trigueros, Cristobal de los Ríos, Manuel Vidal-Sanz

**Affiliations:** ^1^Departamento de Oftalmología, Universidad de Murcia e IMIB-Arrixaca, Murcia, Spain; ^2^Instituto de Investigación Sanitaria, Hospital Universitario de la Princesa, Madrid, Spain; ^3^Departamento de Ciencias Básicas de la Salud, Universidad Rey Juan Carlos, Alcorcón, Spain; ^4^Instituto Universitario de Oftalmobiología Aplicada (IOBA), Retina Group, Universidad de Valladolid, Valladolid, Spain

**Keywords:** NMDA excitotoxicity, glaucoma, retina ganglion cell, neuroprotection, Brn3a + RGCs, SD-OCT, αRGCs, retina calcium blocker

## Abstract

**Purpose:**

The aim of this study was to investigate, the neuroprotective effects of a new Gramine derivative named: ITH12657, in a model of retinal excitotoxicity induced by intravitreal injection of NMDA.

**Methods:**

Adult Sprague Dawley rats received an intravitreal injection of 100 mM NMDA in their left eye and were treated daily with subcutaneous injections of ITH12657 or vehicle. The best dose–response, therapeutic window study, and optimal treatment duration of ITH12657 were studied. Based on the best survival of Brn3a + RGCs obtained from the above-mentioned studies, the protective effects of ITH12657 were studied *in vivo* (retinal thickness and full-field Electroretinography), and *ex vivo* by quantifying the surviving population of Brn3a + RGCs, αRGCs and their subtypes α-ONsRGCs, α-ONtRGCs, and α-OFFRGCs.

**Results:**

Administration of 10 mg/kg ITH12657, starting 12 h before NMDA injection and dispensed for 3 days, resulted in the best significant protection of Brn3a + RGCs against NMDA-induced excitotoxicity. *In vivo*, ITH12657-treated rats showed significant preservation of retinal thickness and functional protection against NMDA-induced retinal excitotoxicity. *Ex vivo* results showed that ITH12657 afforded a significant protection against NMDA-induced excitotoxicity for the populations of Brn3a + RGC, αRGC, and αONs-RGC, but not for the population of αOFF-RGC, while the population of α-ONtRGC was fully resistant to NMDA-induced excitotoxicity.

**Conclusion:**

Subcutaneous administration of ITH12657 at 10 mg/kg, initiated 12 h before NMDA-induced retinal injury and continued for 3 days, resulted in the best protection of Brn3a + RGCs, αRGC, and αONs-RGC against excitotoxicity-induced RGC death. The population of αOFF-RGCs was extremely sensitive while α-ONtRGCs were fully resistant to NMDA-induced excitotoxicity.

## Highlights

- Administration of 10 mg/kg ITH12657, starting 12 hours before NMDA injection and dispensed for 3 days, resulted in the best significant protection of Brn3a+RGCs against NMDA-induced excitotoxicity.
- ITH12657-treatment showed a significant preservation of retinal thickness when compared to vehicle-treatment.
- Treatment with ITH12657 resulted, in smaller reductions of pSTR and b-wave amplitudes compared to those treated with vehicle, indicating that ITH12657 treatment offered functional protection against NMDA-induced retinal excitotoxicity.
- ITH12657 afforded a significant protection against NMDA-induced excitotoxicity for the populations of Brn3a+RGC, αRGC, and αONs-RGC, but not for the population of αOFF-RGC.

## 1 Introduction

The retina has been largely used to explore the response of adult central nervous system (CSN) neurons to injury, protection and regeneration (McKerracher et al., 1990a,b; Whiteley et al., 1998; Aviles-Trigueros et al., 2000; Vidal-Sanz et al., 2002, 2015b, 2017; Lindqvist et al., 2004).

There are several markers that allow the identification of large populations of rodent RGCs (pan-markers) such as Brn3a or RBPMS (Nadal-Nicolas et al., 2023) or specific related RGCs groups, also named subclasses, such as the intrinsically photosensitive RGCs (Vidal-Villegas et al., 2021a,b) or the alfa RGCs (αRGCs; Gallego-Ortega et al., 2022), among others (Tran et al., 2019). Immunohistochemical studies using a combination of markers allow the study, in parallel but independently, of how different RGCs respond to different retinal injuries (Vidal-Sanz et al., 2015a; Agudo-Barriuso et al., 2016; Di Pierdomenico et al., 2022b) and protection (Valiente-Soriano et al., 2015; Rovere et al., 2016; Sanchez-Migallon et al., 2018). For instance, the expression of Brn3a by rodent RGCs has allowed to identification of the main population of RGCs, which accounts for approximately 96% of the RGC population (Nadal-Nicolas et al., 2014).

Recent studies have suggested up to 46 different types of rodent retinal ganglion cells (RGCs), based on various criteria such as their shape, their response to light, their brain connections and the molecular expression of particular genes (Nassi and Callaway, 2009; Sanes and Masland, 2015; Baden et al., 2016; Rheaume et al., 2018; Christensen et al., 2019; Sweeney et al., 2019; Goetz et al., 2022; Huang et al., 2022). Indeed, the use of readily available immunocytochemical markers has allowed to identify the αRGCs, a subclass of RGCs well-characterizaed by their large size, fast conducting velocity and mono-stratified dendritic arbors within different strata of the inner retinal layer (Gallego-Ortega et al., 2021, 2022). Moreover, this class acquires particular relevance because it may constitute the rodent orthologue of the midget RGCs, the most abundant population of RGCs subserving visual acuity and color vision in primates (Hahn et al., 2023). This population in the albino rat accounts for approximately 2.2% of all RGCs (Gallego-Ortega et al., 2021) with four recently described subtypes according to their stratification in the inner layer and functional response to light; ON sustained (αONsRGCs), ON transient (αONtRGCs), OFF sustained (αOFFsRGCs) and OFF transient (αOFFtRGCs; Gallego-Ortega et al., 2022). For the present studies, we identify and quantify αRGCs, αONsRGCs, αONtRGCs and αOFFRGCs.

Glutamate is the main excitatory neurotransmitter in the (CNS), including the retina where it mediates transmission through the main direct pathway, from photoreceptors to bipolars and RGCs have glutamate receptors, including the NMDA receptors, and their overstimulation can cause neuronal excitotoxicity, that is cell death by excessive activation. The NMDA receptors are permeable to calcium and sodium and have important roles in neuronal plasticity, learning and memory. However, excessive NMDA receptor stimulation may result in changes in the Na^+^/K^+^ balance and entry of large amounts of Ca^2+^ into the cell (Manev et al., 1989) which may result in direct damage by activating enzymes that damage DNA and cell membranes (Stavrovskaya and Kristal, 2005) or by inducing apoptosis through activation of cAMP (Hardingham et al., 2002). Glutamate excitotoxicity is believed to play an important role in the loss of RGCs in various retinal injuries (Lucas and Newhouse, 1957; Choi, 1988), such as glaucoma (Dreyer et al., 1996; Vorwerk et al., 2004; Izzotti et al., 2006; Tezel, 2013), transient ischemia (Lam et al., 1997) and optic nerve injury (Schuettauf et al., 2000; Kermer et al., 2001). In addition, can also play a key role in many CNS diseases that involve neuronal death (Almasieh et al., 2012). Animal models of NMDA-induced retinal excitotoxicity are often used to investigate molecular mechanisms of RGC apoptosis and the way to protect them (Kobayashi et al., 2017; Fahrenthold et al., 2018; Pichavaram et al., 2018; Lambuk et al., 2019). The effects of NMDA-mediated excitotoxicity on RGCs have been previously investigated in adult rats (Gomez-Vicente et al., 2015; Vidal-Villegas et al., 2019) and mice (DeParis et al., 2012; Wang et al., 2018). Calcium channel blockade can improve RGC survival after excitotoxicity-induced injury by reducing calcium influx into neurons and preventing cell damage. Calcium channel blockers can act on different types of channels, such as L, T, N or P/Q channels. Examples of calcium channel blockers are nimodipine, nifedipine and verapamil (Chen et al., 2022; Evangeliou et al., 2023).

ITH12657 (1-benzyl-5-methyl-3-(piperidin-1-ylmethyl-1H-indole, 2) is a Gramine derivative that exhibits neuroprotective properties against Alzheimer’s disease (AD; Lajarin-Cuesta et al., 2016; Gonzalez et al., 2018) and represent a hopeful strategy for the treatment of neurodegeneration. Among the mechanisms of action exerted by ITH12657 are the blockade of voltage-dependent calcium channels (VGCC), which prevents excess calcium entry into neurons and associated cell damage, and the prevention of inhibition of protein phosphatase 2A (PP2A), an enzyme that regulates Tau protein phosphorylation of neurofibrillary tangles (Gonzalez et al., 2018).

In this work, we further investigate the response of two different RGC populations, the general Brn3a- and the α-RGCs with their subtypes, to retinal excitotoxicity induced by intravitreal injection of 100 mM NMDA and protection with systemic administration of ITH12567. The purpose of this study in adult albino rats was twofold. In an initial group of experiments, we characterized the protection afforded by ITH12657 on the survival of Brn3a^+^RGCs following a single intravitreal injection of 5 μL containing 100 mM NDMA. These studies indicated that the best dose, time window and length of treatment regime for ITH12657 were 10 mg/kg, starting 12 h before intravitreal NMDA injection and maintained for 3 additional days following intravitreal NMDA. A second group of experiments further investigated the neuroprotective effects of ITH12657 on the retina, and this was assessed longitudinally *in vivo* with morphological and functional techniques, and *ex vivo* by counting and mapping in retinal whole-mounts total numbers of surviving Brn3a^+^RGCs, αRGCs and their subtypes (αONsRGCs, αONtRGCs or αOFFRGCs).

## 2 Methods

### 2.1 Animal handling

Adult female albino Sprague Dawley (SD; *n* = 144) rats aged 3 months and weighing approximately 180–220 g were used since there is sufficient evidence in the literature to indicate that data obtained from female rodents are no more variable than those from male rodents (Becker et al., 2016; Beery, 2018), for a better comparison with previous published work, and their better docility and smaller size. The rats were bred and maintained in the Experimental Animal Facility of the University of Murcia under controlled light (12-h light–dark cycles with light intensity within the cages ranging from 5 to 30 lx) and temperature conditions (23–24°C), and with access to food and water *ad libitum*.

The animals were treated according to the current European and national regulations and, specifically, according to Directive 86/609/EEC, 2010/63/EU on the protection of animals used for scientific purposes, the R.D.1201/2005 on the protection of animals used for experimental and other scientific purposes, the Law 32/2007 for the care of animals, in their exploitation, transport, experimentation, and slaughter, the Association for Research in Vision and Ophthalmology (ARVO) guidelines for the use of animals in ophthalmic and visual system experimentation. Animal experiments were approved by the University of Murcia Ethical animal studies committee (Protocols A13171103, A13170110 and A13170111).

### 2.2 Experimental design

**Figure 1:**
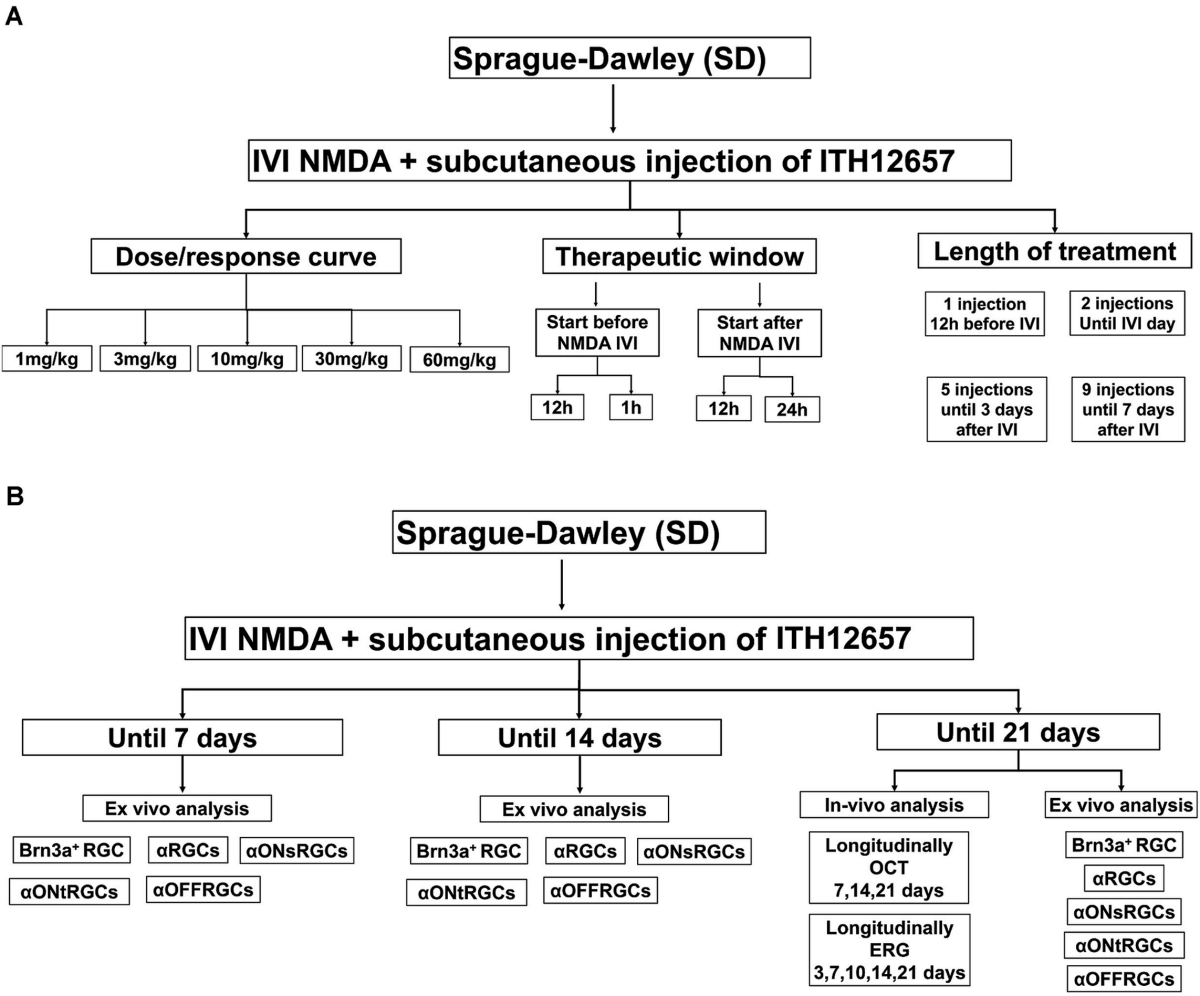
Experimental design of the analysis of the protective effects of the compound ITH12657 in an NMDA-induced retinal excitotoxicity model. **(A)** The first group (*n* = 96) was used to determine the best dose, therapeutic window, and treatment regime of ITH12657. **(B)** The second group (*n* = 48) was designed to investigate ITH12657-afforded protection, longitudinally *in vivo* and by quantifying the survival of different RGC types at 7, 14 or 21 days.

Rats were divided into two main groups of animals. A first group (*n* = 96) was used to determine the best dose, therapeutic window and regime of ITH12657 treatment, that is, to: (i) characterize the dose–response curve (*n* = 36) (6 subgroups) with different concentrations of ITH12657 (*n* = 6 per subgroup), sacrificed at 7 days after NMDA-induced excitotoxicity: vehicle, 1, 3, 10, 30, or 60 mg/kg; (ii) determine the therapeutic window of ITH12657 (*n* = 30) with 5 subgroups depending on the time of treatment initiation (*n* = 6 per subgroup), sacrificed 7 days after NMDA-induced excitotoxicity: 12 h before, 1 h before, 12 h after, 24 h after or vehicle; and (iii) to investigate the optimal treatment duration (*n* = 30), ITH12657 was administered for 1, 2, 3, or 7 days after NMDA injection (Figure 1A). The second group (*n* = 48) was used to investigate the ITH12657 afforded protection longitudinally *in vivo* using functional (full-field ERG was recorded at 3, 7, 10, 14, and 21 days) and morphological analysis [Optical coherence tomography (OCT) was performed at 7, 14, and 21 days] (*n* = 16) and *ex vivo* by quantifying at different periods of 7, 14 or 21 days the survival of several RGC types; the general population of Brn3a^+^RGCs, the population of αRGCs (OPN^+^RGCs) and their subtypes αONsRGCs (OPN^+^Tbr2^+^RGCs), αONtRGCs (OPN^+^Brn3a^−^Tbr2^−^RGCs) and αOFFRGCs (OPN^+^Brn3a^+^RGCs). Two subgroups (one treated with vehicle, and one treated with ITH12657 10 mg/kg) (*n* = 8 per subgroup) were analyzed at 7, 14 or 21 days after NMDA-induced excitotoxicity. The group analyzed at 21 days (*n* = 16) was used for longitudinal analysis of OCT and full-field electroretinography (ERG; Figure 1B).

### 2.3 NMDA-induced excitotoxicity

Animals were placed under general anesthesia and received an intravitreal injection (IVI) of either 5 μL of NMDA (100 mM) or saline into their left eye, while the right eye served as an untreated intact control. A 30G needle was used to create a puncture approximately 1 mm posterior to the limbus, through which a 32G Hamilton syringe needle was inserted and advanced toward the center of the globe, taking care to avoid lens damage. Any subjects that experienced retinal injury or cataract formation during the intravitreal injection were excluded from the study.

### 2.4 Administration of ITH12657

Animals received a daily subcutaneous (sc) injection of 1 mL of either ITH12657 or its vehicle (saline with 1% DMSO) for 3 days. The ITH12657 molecule was synthesized according to previously established procedures and provided by Gonzalez et al. (2018).

### 2.5 *In vivo* analysis

#### 2.5.1 Optical coherence tomography (OCT)

The retinal thickness was analyzed longitudinally *in vivo* using a Spectralis SD-OCT system (Heidelberg, Germany) specifically adapted for its use in rat eyes (Rovere et al., 2015; Nadal-Nicolas et al., 2018). This group of animals (*n* = 16) was treated with ITH12657 10 mg/kg (*n* = 8) or vehicle (*n* = 8) and analyzed at 7, 14 and 21 days after NMDA excitotoxicity. At the end of the OCT analysis, this group which was also assessed functionally, was used for *ex vivo* analysis of RGC survival. General anesthesia was administered and 1% tropicamide drops (Colicursi tropicamide 1%; Alcon-Cusí, S.A., Barcelona, Spain) were instilled in both eyes to induce mydriasis. Imaging was performed using two options: 31 B-scans (240-micron area) to quantify total and outer retinal thickness and the peripapillary ring B-scan for Ganglion Cell Complex (GCC) thickness.

To measure inner and total retinal thickness we followed previously described methods that are standard in the laboratory (Ortin-Martinez et al., 2014; Rovere et al., 2015; Vidal-Villegas et al., 2019). In brief, four measurements of total (from the nerve fiber layer to the retinal pigment epithelium) and inner (from the inner limiting membrane to the outer boundary of the inner nuclear layer) thickness were taken on three lines of the scans (superior, inferior, and through the optic nerve). On the upper and lower lines, these measurements were taken at four equidistant points, while on the central line, which includes the optic disc, two measurements were taken on either side of the optic disc. This resulted in a total of 12 measurements (4 measurements on 3 lines) of total and inner thickness per subject.

#### 2.5.2 Electrophysiology

Full-field Electroretinography (ERG) was performed according to previously published methods (Alarcon-Martinez et al., 2009, 2010; Gallego-Ortega et al., 2020, 2021). Briefly, after 12 h of dark adaptation, rats were anaesthetized and both eyes were dilated with topical mydriatic (Tropicamida 1%; Alcon-Cusí, S.A. Barcelona, Spain). Scotopic and photopic responses were recorded simultaneously in both eyes using Burian–Allen corneal bipolar electrodes. A drop of methylcellulose (Methocel^®^ 2%; Novartis Laboratories CIBA Vision, Annonay, France) was applied between the cornea and the electrodes to improve signal conductivity. The reference electrode was placed in the mouth and a needle at the base of the tail served as a ground electrode. Retinal ganglion cell (RGC)-mediated responses were recorded using light flashes ranging from − 4.4 log cd·s/m^2^ scotopically, rod-mediated responses were recorded at − 2.5 log cd·s/m^2^, while mixed (a- and b-waves) responses were recorded at − 0.5 log cd·s/m^2^. Cone-mediated responses were elicited using 0.5 log cd·s/m^2^ flashes on a 30 cd/m^2^ rod-saturating background. Electrical signals were digitized at 20 kHz using a PowerLab data acquisition board (AD Instruments, Chalgrove, UK) and standard ERG waves were analyzed according to the guidelines of the International Society for Clinical Electrophysiology of Vision (ISCEV). A total of 16 rats were used for the electrophysiological studies, these were divided into two groups treated with ITH12657 (*n* = 8) or vehicle (*n* = 8) and analyzed longitudinally at 3, 7, 10, 14 or 21 days. This group had also been analyzed *in vivo* for OCT. At the end of these studies, this group was used for *ex vivo* histological RGC survival analysis at 21 days.

### 2.6 *Ex vivo* analysis

#### 2.6.1 Identification of different RGC populations

**Figure 2:**
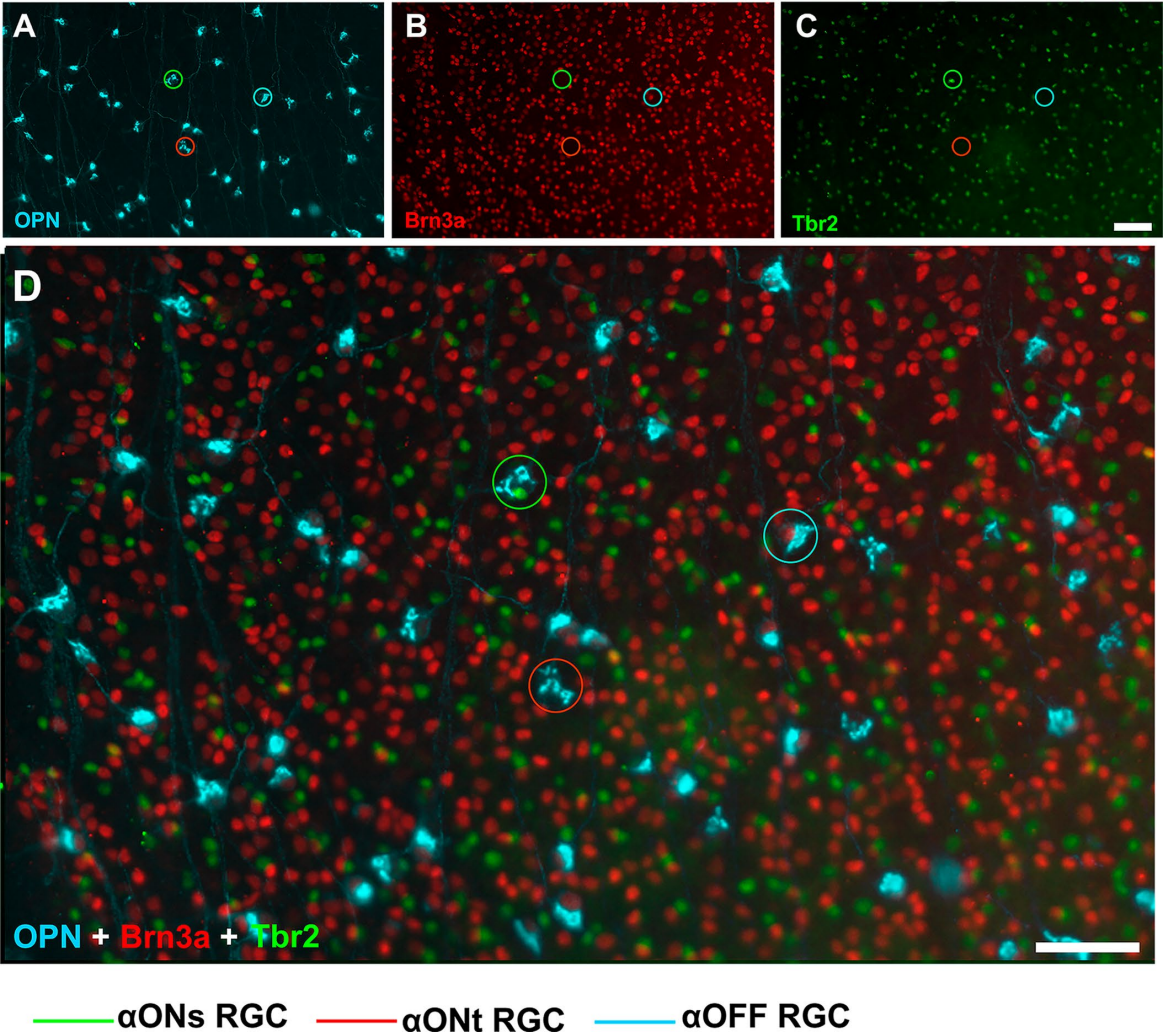
Immunohistochemical detection of αRGCs populations. Representative fluorescence micrographs of osteopontin **(A)**, Brn3a **(B)**, Tbr2 **(C)**, and the merge **(D)** colocalization of RGC from control animals. Scale bar, 50 μm.

In order to study the idiosyncratic responses to injury-induced retinal degenerations and neuroprotection of RGCs, we analyse few types of RGCs for which immunoystochemical tools are available. The population of Brn3a expressing RGCs, were immunostained with Brn3a antibodies (Mouse anti-Brn3a, MAB1585 Millipore, Merck Burlington, MA, USA). To identify the alpha RGCs (αRGCs) and their subtypes ON sustained (αONsRGCs), ON transient (αONtRGCs), and OFF (αOFFRGCs), all retinas were exposed to various combinations of antibodies and subsequently different wavelength fluorophore-conjugated secondary antibodies following recently described methods (Gallego-Ortega et al., 2021, 2022). In brief: (i) antibodies against osteopontin (OPN) (Goat anti-Osteopontin, AF808 Biotech, Biotech Spain, Barcelona, Spain) were used to detect the αRGC population, (ii) colocalization of OPN and Tbr2 (T-box transcription factor T-brain 2) (Rabbit anti-Tbr2, AB23345 Abcam, Cambridge, UK) was used to detect αONsRGCs, (iii) colocalization of OPN and Brn3a was used to detect αOFFRGCs, and (iv) positive signal for OPN but negative for Brn3a and Tbr2 was used to detect αONtRGCs (Figure 2).

#### 2.6.2 Image analysis

Immunostained retinal whole-mounts were analyzed and imaged using an epifluorescence microscope (Leica DM6-B; Leica Microsystems, Wetzlar, Germany) according to previously described methods (Gallego-Ortega et al., 2022; Di Pierdomenico et al., 2022a). Briefly, to reconstruct retinal flat mounts, multiple frames were acquired for each filter in a contiguous raster scan pattern (x10) with no overlap or spacing between images. Individual images were focused before acquisition and obtained using the same focus for each specific filter. To acquire higher magnification images of the retina, x40 and x60 objectives were used.

#### 2.6.3 Quantification and co-expression analysis

To determine in flat-mounted retinas the total number of RGCs immunolabeled with Brn3a, we used standard computer routines developed in our laboratory (Ortin-Martinez et al., 2015; Nadal-Nicolas et al., 2018). To count αRGCs and different αRGC subtypes, the labeled and/or colocalized cells were manually marked on each retinal photomontage and the total number of marks per retina was quantified using Image ProPlus software (IPP 5.1 for Windows; Media Cybernetics, Silver Spring, MD, USA) as previously described (Gallego-Ortega et al., 2022).

#### 2.6.4 Topographical distributions

The topographic distribution of Brn3a^+^RGCs, αRGCs and αRGC subtypes was studied using isodensity or neighbor maps according to previously described methods (Salinas-Navarro et al., 2009; Galindo-Romero et al., 2013; Valiente-Soriano et al., 2014; Gallego-Ortega et al., 2022). All maps were plotted using SigmaPlot software (SigmaPlot 11.0 for Windows; Systat Software, Inc., Richmond, CA, USA) and a color code was used to represent the data.

#### 2.6.5 Statistic

RGC numbers are expressed as mean ± standard deviation (SD) of the mean. Statistical comparisons between different groups were done using one-way analysis of variance (ANOVA-Tukey’s *Post hoc* tests), and comparisons between two groups were done using Student’s *t*-test with the software Graph Pad Prism^®^ for Windows (Version 5.01; GraphPad Software Inc., La Jolla, CA, EEUU). A value of *p* ≤ 0.05 was considered statistically significant.

## 3 Results

### 3.1 ITH12657 dose, time window and length of treatment

The first group of experiments was designed to characterize the dose/response curve, therapeutic window, and neuroprotective effects of ITH12657 as a neuroprotectant for RGCs against NMDA-induced excitotoxicity. These studies indicated that 10 mg/kg ITH12657, starting 12 h before intravitreal NMDA injection and maintained for 3 additional days afforded the best protection on the survival of Brn3a^+^RGCs following a single intravitreal injection of 100 mM NMDA.

#### 3.1.1 ITH12657 dose–response

To analyze the protective effects of ITH12657, we determined the lowest concentration where the best results were obtained. Following intravitreal injection in the left eye of (5 μL of 100 mM NMDA) or vehicle, different doses of the compound ITH12657 were administered (1, 3, 10, 30 or 60 mg/kg) (*n* = 6 per group), and retinas were examined at 7 days for Brn3a^+^RGC survival.

**Figure 3:**
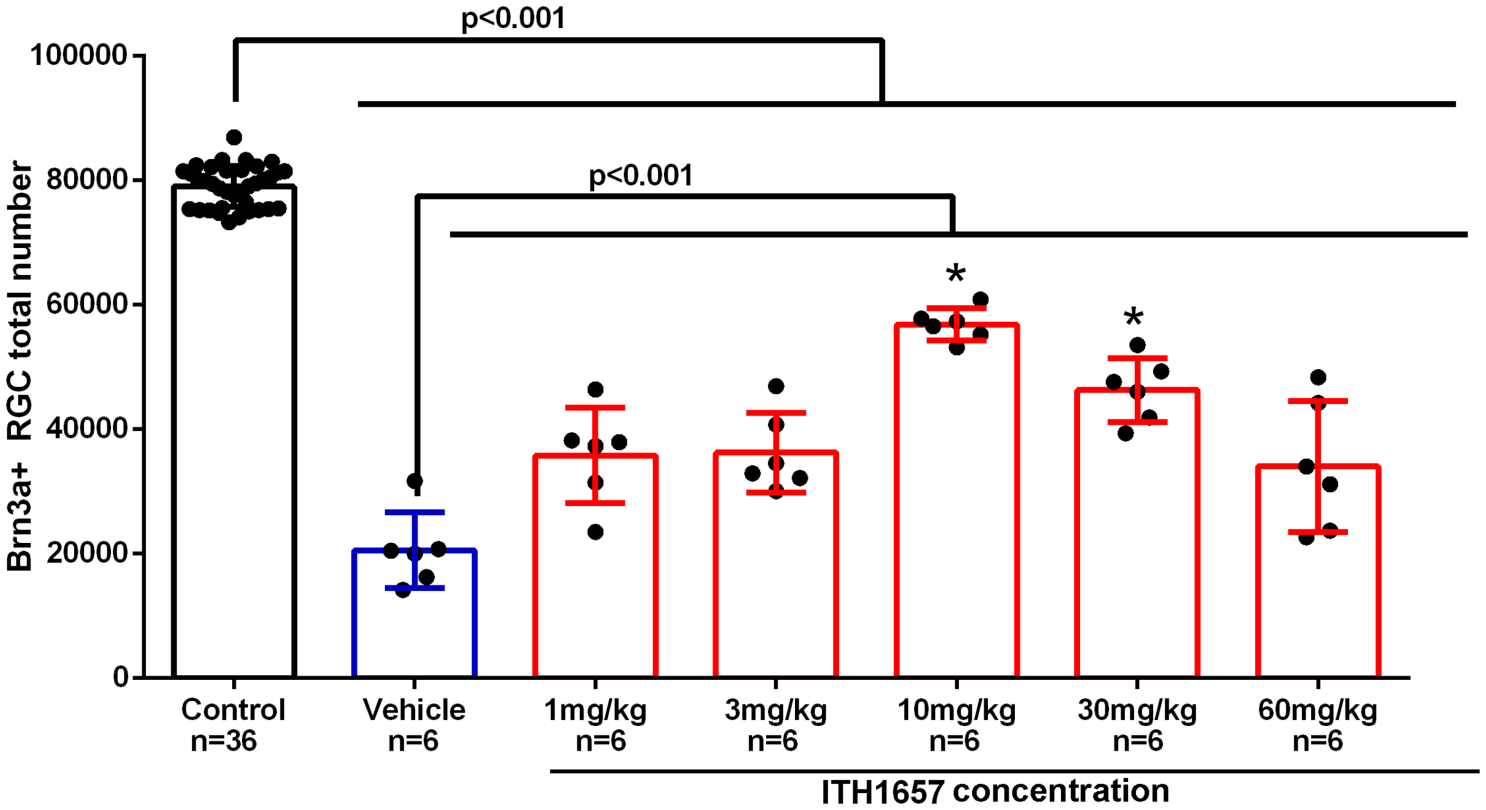
Dose–response curve. Histogram showing total Brn3a^+^RGCs surviving after intravitreal injection (IVI) of NMDA-induced excitotoxicity and subcutaneous treatment with different doses of the compound ITH12657. The survival rate at 7 days was higher in the group in which ITH1657 was administered at a dose of 10/mg/kg. *Significant differences *p* < 0.05 (One-way ANOVA).

All experimental groups showed differences when compared with the control (intact right eyes) group (One-way ANOVA, *p* < 0.001) (Figure 3) indicating that intravitreal injection (IVI) of NMDA resulted in substantial Brn3a^+^RGC loss (28% survival) (Figure 3). The ITH12657-treated groups showed significantly greater total numbers of surviving Brn3a^+^RGCs when compared to the vehicle-treated ones, thus showing effective ITH12657 afforded protection against 100 mM NMDA-induced excitotoxicity (Figure 3). Moreover, the 10 mg/kg ITH12657-treated group showed higher survival than the 3 or 30 mg/kg ITH12567-treated groups. Thus, for the rest of the study we used the ITH12567 10 mg/kg dose that resulted in the highest (72%) survival of Brn3a^+^RGCs (Figure 3).

#### 3.1.2 Therapeutic window of ITH12657

We next investigated the therapeutic window for compound ITH12657. Five groups of animals (*n* = 6 per group) were injected with NMDA in the left eye while the right eye was used as control, and vehicle (daily injection) or ITH12657 was administered (i.p.) with different time regimes: (i) 12 h before, just before and the following 6 days; (ii) 1 h before and the following 6 days; (iii) 12 h after and the following 6 days and; and (iv) 24 h after and the following 5 days.

**Figure 4:**
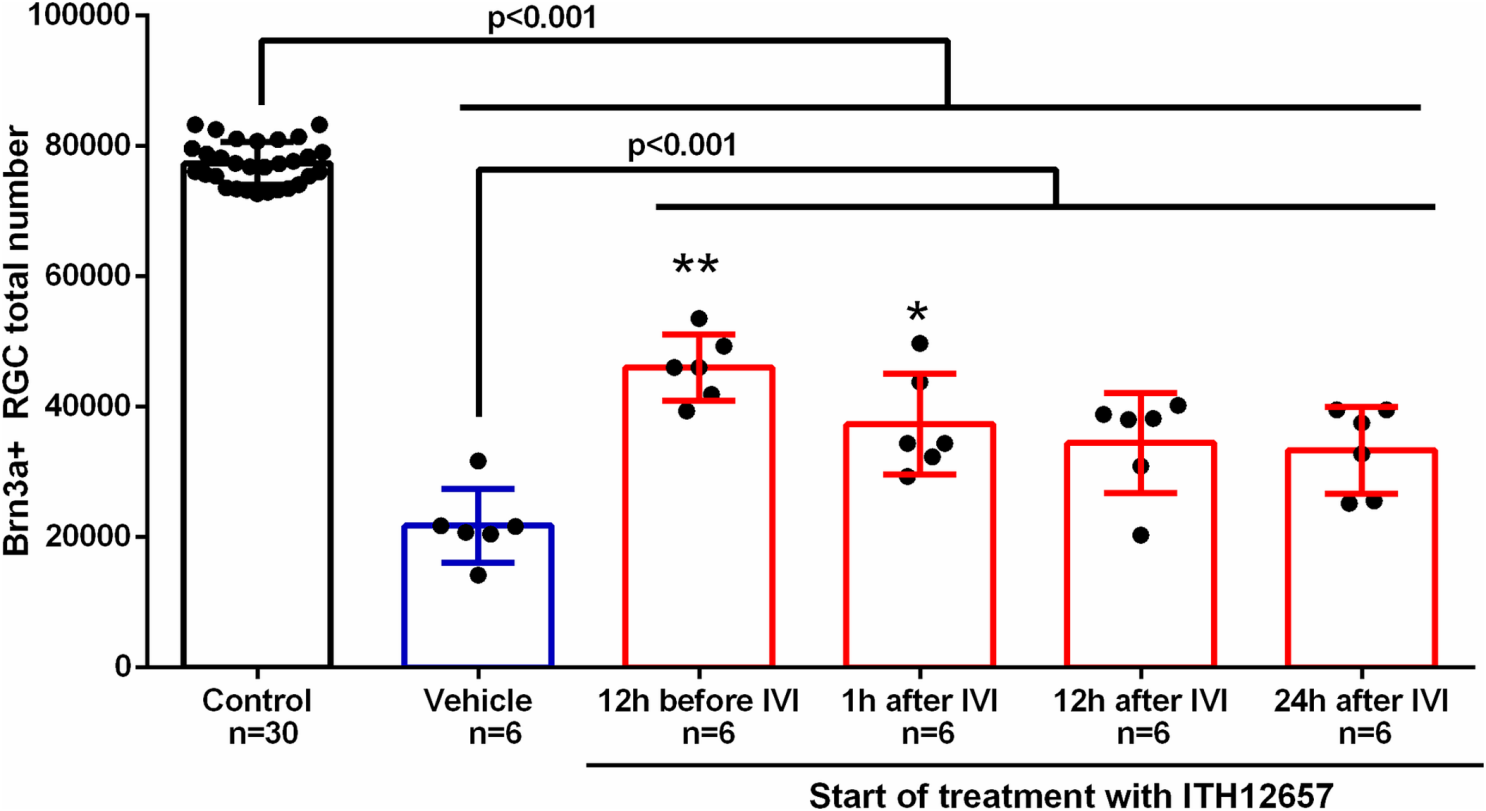
Therapeutic window. Histogram of the study of the therapeutic window of the administration of vehicle or the compound ITH12657 after intravitreal injection (IVI) of 5 μL of 100 mM NMDA in the left eye. The survival rate was highest in the group in which treatment started 12 h before excitotoxicity. One-way ANOVA analysis significant differences **p < 0.0001 and *p < 0.05.

As for the previous dose–response study, we found that all experimental (left eye) groups presented significant differences in total numbers of surviving Brn3a^+^RGCs when compared to the control (right eye) group (One-way ANOVA, *p* < 0.0001), and all ITH12657-treated groups showed significantly greater total numbers of Brn3a^+^RGCs when compared to the vehicle-treated groups (One-way ANOVA, *p* < 0.0001; Figure 4). The group treated 12 h before, just before and the following 6 days after NMDA intravitreal injection, showed the highest Brn3a^+^RGC survival, when compared to the other groups. The group treated with ITH12657 1 h after NDMA-induced excitotoxicity showed no significant differences with the group treated 12 h later (34,448 ± 7,647 Brn3a^+^RGC) or 24 h later (33,363 ± 66.53 Brn3a^+^RGC) (Figure 4). Thus, overall, the best protective effects were obtained when the ITH12657 treatment started 12 h prior to NMDA intravitreal injection.

#### 3.1.3 Length of ITH12657 treatment

Once the ideal concentration (10 mg/kg) and therapeutic window (12 h before intravitreal injection of NMDA) were found, we determined the optimal length of ITH12657 treatment. For this purpose, four groups of animals (*n* = 6 per group) were injected with NMDA in the left eye while the right eye was used as control and ITH12657 was injected sc. 12 h before intravitreal injection of NMDA, but for different lengths of time: (i) group 1 received a single injection 12 h prior to NMDA injection, (ii) group 2 received treatment 12 h prior and just prior to NMDA injection (2 injections), (iii) group 3 received treatment 12 h prior, just prior to NMDA injection, and every day until day 3 post-NMDA injection (5 injections), and (iv) group 4 received treatment until 7 days post-NMDA injection (9 injections).

**Figure 5:**
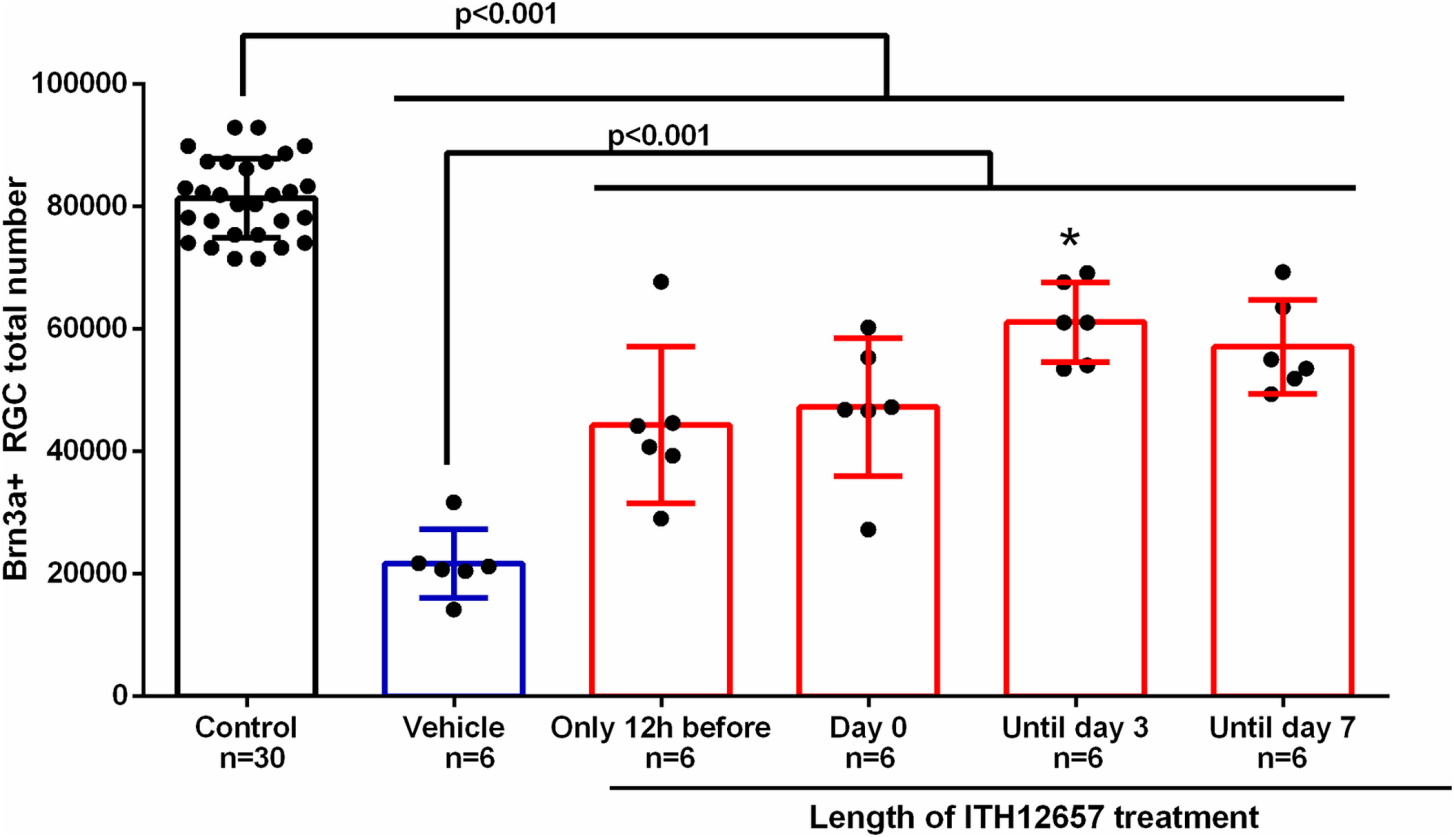
Length of treatment. Histogram of the study of the length of treatment of the administration of vehicle or the compound ITH12657 after intravitreal injection of 5 μL of 100 mM NMDA or vehicle in the left eye. The survival rate was higher in the group in which treatment started 12 h prior to NMDA intravitreal injection and was maintained until day 3. *Significant differences *p* < 0.0001 (One-way ANOVA).

All ITH12657 treated groups exhibited significant differences in mean total numbers of Brn3a^+^RGCs when compared to the control (right eyes) (One-way ANOVA, *p* < 0.0001) or the vehicle-treated groups of eyes (One-way ANOVA, *p* < 0.0001) (Figure 5). Quantification of Brn3a^+^RGCs in ITH12657-treated groups revealed that animals treated until 3 days post intravitreal injection of NMDA showed the highest survival when compared to the other groups (One-way ANOVA, *p* < 0.04) (Figure 5).

### 3.2 *In vivo* and *ex vivo* assessment of ITH12657-afforded retinal protection against NMDA-induced excitotoxicity

In a second group of experiments, we employ *in vivo* functional and morphological techniques, as well as *ex vivo* immunohistochemical methods to assess the effects of intravitreal NMDA-induced excitotoxicity and protection afforded with systemic ITH12657 in the adult rat retina. We assessed quantitatively in the same retinal whole-mounts the survival of Brn3a^+^RGCs, αRGCs, α-ONs-RGCs, α-ONt-RGCs and α-OFF-RGCs, at 7, 14 or 21 days. These studies indicated that ITH12657 afforded morphological and functional longitudinal protection of the retina. Moreover, the quantitative analysis of RGCs indicate that ITH12657 afforded significant protection for Brn3a^+^RGCs, αRGCs and αONsRGCs, but not for αOFFRGCs, while the αONtRGCs were fully resistant to NMDA-induced retinal injury.

#### 3.2.1 *In vivo* study of retinal thickness. ITH12657-afforded protection against NMDA-induced excitotoxicity

**Figure 6:**
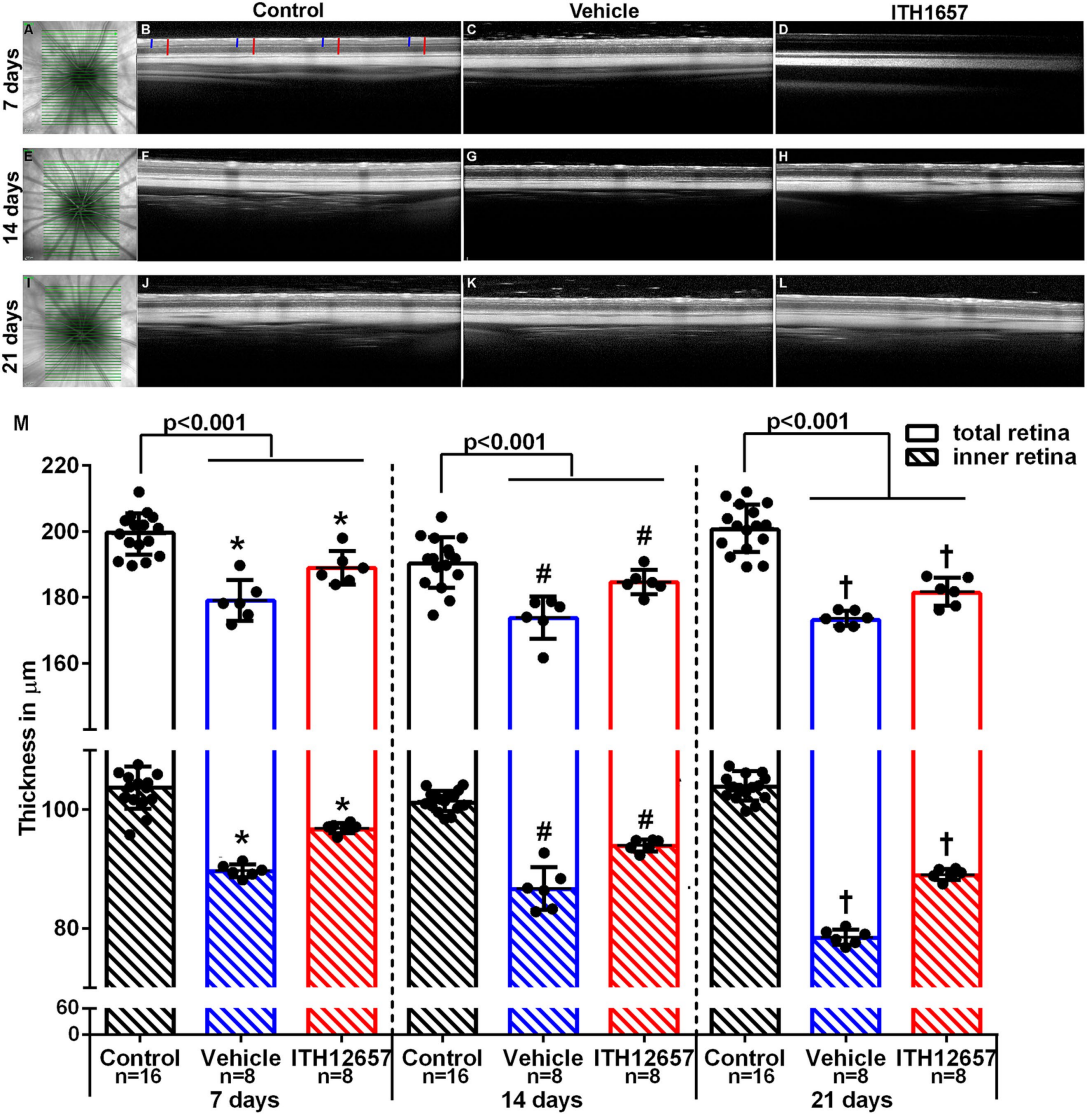
Retinal thickness. *In vivo* images obtained by optical coherence tomography (SD-OCT) of total and inner retinal thickness. Representative images of the fundus **(A,E,I)**. Representative images of the contralateral **(B,F,J)** and experimental retinal layers, analyzed at 7, 14 or 21 days after NMDA injection treated with vehicle **(C,G,K)** or ITH12657 **(D,H,L)**. In panel **(B)**, the red lines show the measurements made in the total retina and the blue lines show the inner retina measurements. **(M)** Histograms showing total and inner retinal thickness in control (right) (*n* = 16 for each time point), and in experimental (left) retinas treated with vehicle (in blue) or ITH12657 (in red) at 7 (*n* = 8), 14 (*n* = 8), and 21 (*n* = 8) days after intravitreal injection of 100 nM NMDA in the left eye. Student’s *t*-test statistical significance *p < 0.001, ^#^*p* < 0.001, or ^†^*p* < 0.001.

The results obtained *in vivo* with SD-OCT showed that total and inner retinal thickness were reduced significantly in all experimental groups when compared to the control group, with retinal thinning mainly due to inner retina reduction. Total retinal thickness in the NMDA-injected retinas was significantly lower when compared to the control group (intact right retinas) at all time intervals studied, with an average thinning of 10.3, 8.65 and 13.65% at 7, 14 and 21 days, respectively (Figure 6). Furthermore, retinal thickness was significantly higher in the retinas of the ITH12657-treated animals than in the retinas of the vehicle-treated animals; the vehicle-treated retinas showed an inner retinal thickness reduction of 13.6, 9.46% or 24.54% at 7, 14 or 21 days, respectively (Figure 6), while the retinas of ITH12657-treated animals showed reductions of 6.8, 8.2% or 14.4% at 7, 14, or 21 days, respectively (Student’s *t*-test, *p* < 0.001) (Figure 6). Overall, these results indicate that ITH12657 treatment significantly reduced NMDA-induced thinning of the retina, acting mainly on the inner layers.

#### 3.2.2 Retinal functional protection with ITH12657 after NMDA-induced excitotoxicity

To analyze the effects of NMDA-induced retinal excitotoxicity and to investigate whether ITH12657 affords functional protection of the retina, we recorded longitudinally full-field electroretinograms analyzing the pSTR (produced by RGCs), the a-wave (produced by photoreceptors) and the b-wave (produced by cone bipolar cells, CBs) of the mixed response (Gallego-Ortega et al., 2020). For this analysis, we employed the same group of 16 animals used for the SD-OCT longitudinal analysis, and recordings were obtained at 3, 7, 10, 14, and 21 days after NMDA injection.

**Figure 7:**
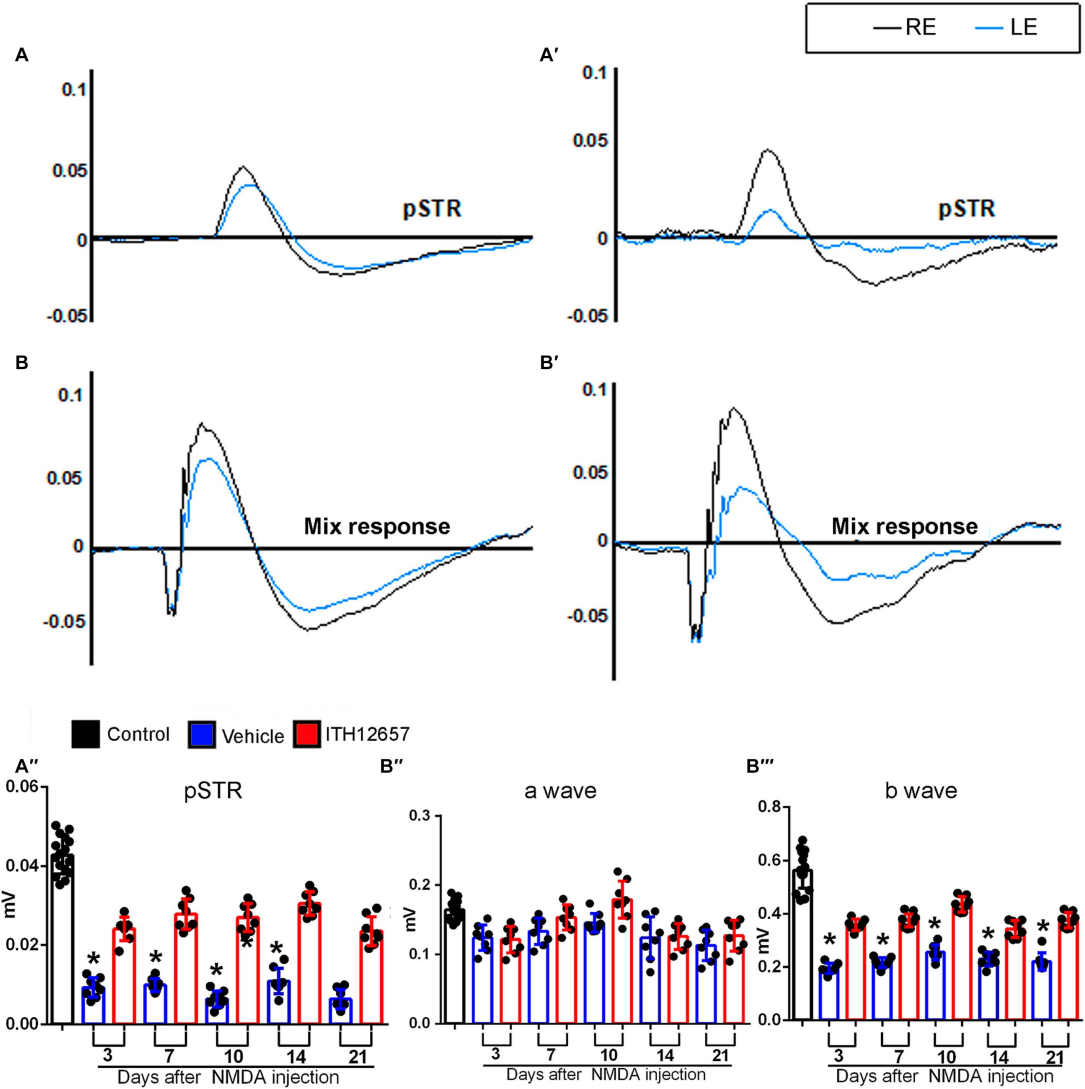
Functional study of the neuroprotection afforded by ITH12657 after intravitreal injection of NMDA in the left eye. **(A,A’,B,B’)** Representative traces of the control (right intact) (in black) and the experimental (left) eye (in blue) 21 days after received NMDA injection and were treated with ITH12657 **(A,B)** or vehicle **(A’,B’)**. The left column shows representative traces of the positive scotopic threshold response (pSTR) **(A)** and mixed **(B)** waves of the animals that were treated with ITH12657, while the right column shows representative traces of the pSTR **(A’)** and mixed **(B’)** waves in animals treated with vehicle after NMDA excitotoxicity. **(A”,B”’)** Histograms of waveform analysis in control (right intact) (*n* = 16) (in black) or experimental (left) eyes 3, 7, 10, 14 or 21 days after intravitreal injection of NMDA in the left eye, and treatment with ITH12657 (in red) (*n* = 8) or vehicle (in blue) (*n* = 8). RE, right eye; LE, left eye.

Retinal function in the left experimental eyes was impaired following intravitreal injection of NMDA (Figure 7). There were significant differences between the mean amplitudes of the pSTR in the contralateral (intact right) versus the experimental (left) eyes that received an intravitreal injection of NMDA and those treated with vehicle. Three days after NMDA administration, there was an 80% reduction (0.042 ± 0.006 control vs. 0.008 ± 0.0034 vehicle 3d) (One-way ANOVA, *p* < 0.001) in the mean pSTR amplitude of animals receiving vehicle as treatment when compared to control. The decrease in the mean amplitude of the pSTR was maintained throughout the period of study (0.008 ± 0.0034 vehicle 3d vs. 0.01 ± 0.04 vehicle 21d) (One-way ANOVA, *p* > 0.05) (Figure 7).

However, the reduction in mean pSTR amplitude was significantly smaller in animals that were treated with ITH12657 than in those treated with vehicle, at all time-intervals studied (Figure 7), suggesting that ITH12657 affords functional permanent protective properties on RGC functionality after NMDA-induced excitotoxicity. A very similar pattern was observed in the b-wave of the mixed response. The left retinas presented significant differences when compared with the amplitude of the control (right) retinas. However, such reduction in the vehicle-treated group was greater (0.55 ± 0.11 control vs. 0.18 ± 0.07 vehicle 3d) than in the ITH12657-treated one (0.36 ± 0.008 ITH12657 3d). The b-wave amplitudes of the animals that had received the vehicle showed an initial reduction of 68%, whereas those treated with ITH12657 were reduced by only 35%, with no further progression during the rest of the study (One-way ANOVA, *p* < 0.01; Figure 7). The a-wave of the mixed response, which assesses the functional status of photoreceptors, did not show significant changes in any of the study groups or time-intervals analyzed after NMDA-induced excitotoxicity (One-way ANOVA, *p* > 0.05; Figure 7). Thus, it is tempting to suggest that intravitreal injection of NMDA does not result in photoreceptor damage that can be registered functionally with ERG shortly, up to 21 days after injury.

#### 3.2.3 *Ex vivo* analysis of the neuroprotective effects of ITH12657 on Brn3a^+^RGCs after NMDA-induced excitotoxicity

**Figure 8:**
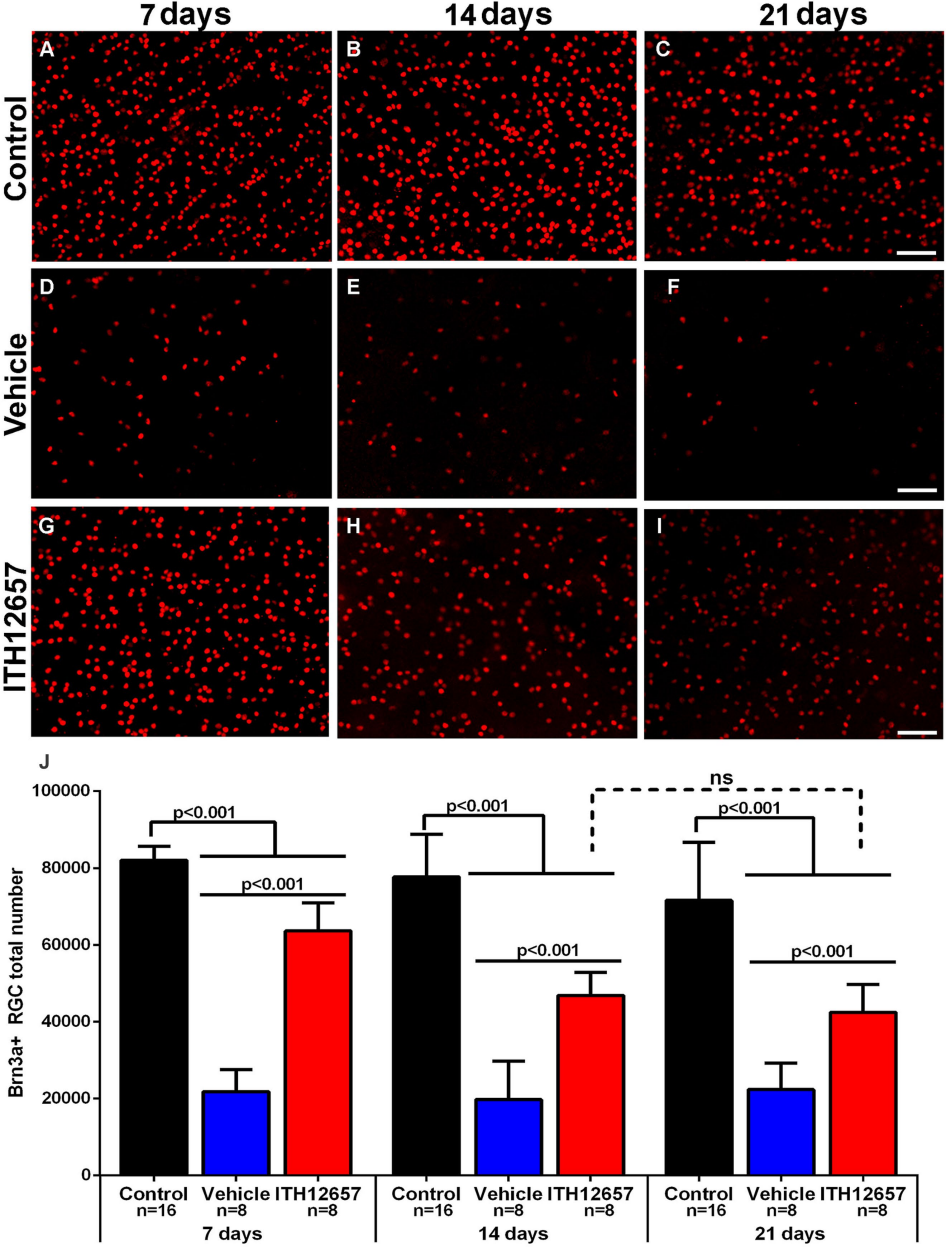
Brn3a^+^RGCs. Representative fluorescence micrographs of Brn3a-immunodetected RGC from control **(A–C)**, and experimental retinas with intravitreal injections of NMDA that were treated with vehicle **(D–F)** or ITH12657 **(G–I)** and examined at 7, 14, or 21 days after NMDA excitotoxicity. **(J)** Histogram (mean ± SD) of the total Brn3a^+^RGC population in control (right intact) (black columns) and experimental (left, injected with NMDA) eyes treated with vehicle (blue columns) or ITH12657 (red columns). ns, not statistically difference, *p* > 0.05 One-way ANOVA. Scale bar, 50 μm.

NMDA-induced excitotoxicity results in the degeneration of the Brn3a^+^RGC population, as already described (Vidal-Villegas et al., 2019; Milla-Navarro et al., 2021). One purpose of the present studies was to investigate the neuroprotective effects of systemically administered ITH12657 against NMDA-induced excitotoxicity, in the Brn3a^+^RGC population. The retinas from the groups of animals treated with vehicle underwent a rapid and massive loss of Brn3a^+^RGCs, already evident by 7 days after NMDA intravitreal injection, with a reduction to 73% of its original value when compared to control retinas (82,061 ± 3,584 control vs. 21,794 ± 57,222 vehicle 3d) (One-way ANOVA, *p* < 0.05). Such reductions were maintained at 14 (19,556 ± 5,119) and 21 days (21,794 ± 5,080) with no significant difference between them (One-way ANOVA, *p* > 0.05) suggesting that following the initial loss of Brn3a^+^RGCs observed by 7 days, there was no further loss (Figure 8; Table 1). Treatment with ITH12657 significantly prevented NMDA-induced degeneration of Brn3a^+^RGCs. When total numbers of Brn3a^+^RGCs were compared between the ITH12657-treated groups versus vehicle-treated groups, significantly greater differences were obtained at all time intervals analyzed with a survival of 78, 69% or 58% of the Brn3a^+^RGC population at 7, 14 or 21 days, respectively (One-way ANOVA, *p* < 0.05; Figure 8; Table 1). When the ITH12657 treated groups were compared at 7 (63,639 ± 7,274) and 14 (56,744 ± 5,995) days, there were significant differences (One-way ANOVA, *p* < 0.05), but no significant differences were found between the 14 and the 21 days groups (56,744 ± 5,995 vs. 47,450 ± 7,255) (One-way ANOVA, *p* > 0.05), suggesting that the ITH12657 afforded protection at 14 days was maintained (Figure 8J; Table 1).

**Table 1:** ITH12657-afforded protection on different RGC types.

	Days after NMDA injection		
	7 days	14 days	21 days
**Brn3a** ^+^ **RGCs**			
Control (*n* = 16)	82,061 ± 3,584	77,677 ± 11,163	71,560 ± 15,129
Vehicle (*n* = 8)	21,794 ± 5722*	19,785 ± 9953*	22,349 ± 6857*
ITH12567 (*n* = 8)	63,639 ± 7274*‡	46,879 ± 5995*‡†	42,450 ± 7274*‡
**αRGC**			
Control (*n* = 16)	2,176 ± 155	2,177 ± 143	2,135 ± 152
Vehicle (*n* = 8)	559 ± 90*	474 ± 94*†	357 ± 127*
ITH12567 (*n* = 8)	1,294 ± 242*‡	832 ± 134*‡	729 ± 192*‡
αONsRGC			
Control (*n* = 16)	1,100 ± 55	1,078 ± 51	1,099 ± 58
Vehicle (*n* = 8)	301 ± 73*	329 ± 13*	295 ± 36*
ITH12567 (*n* = 8)	569 ± 38*‡	512 ± 53*‡	479 ± 75*‡
**αOFFRGC**			
Control (*n* = 16)	789 ± 59	809 ± 61	811 ± 56
Vehicle (*n* = 8)	12 ± 6*	1 ± 0.8*†	3 ± 2*
ITH12567 (*n* = 8)	18 ± 13*	18 ± 16*‡	16 ± 23*‡
**α-ONtRGC**			
Control (*n* = 16)	300 ± 12	289 ± 10	276 ± 14
Vehicle (*n* = 8)	309 ± 37	285 ± 44	292 ± 63
ITH12567 (*n* = 8)	310 ± 33	310 ± 70	260 ± 49

Total numbers of Brn3a + RGC, αRGC, αONsRGC, αOFFRGC or αONtRGC (mean ± SD) in control (contralateral right intact) and experimental (left injected with NMDA) retinas analyzed 7, 14, and 21 days after injection in the left eye of 5 μL containing 100 mM NMDA and daily s.c treatment with vehicle or ITH12567 (10 mg/kg).

* Statistically significant difference with the control group at the same time point. Student’s *t*-test *p* < 0.001.

‡ Statistically significant difference with the vehicle treated group at the same time point. Student’s *t*-test *p* < 0.001.

† Statistically significant difference with previous survival interval. One-way ANOVA *p* < 0.001.

**Figure 9:**
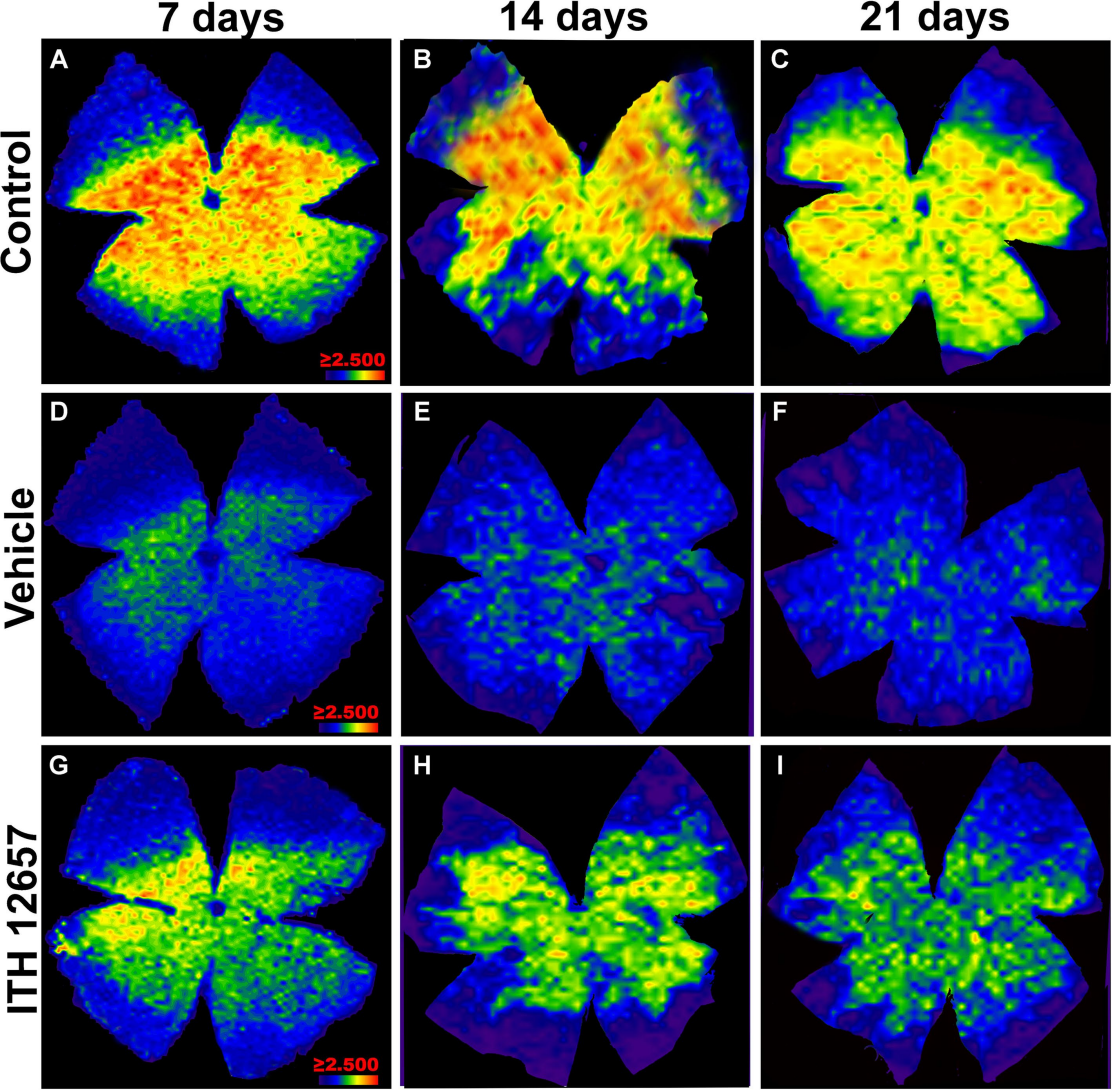
Representative images of the distribution of Brn3a^+^RGCs. Isodensity maps of the retinas of the control group **(A–C)** and experimental group treated with vehicle **(D–F)** or ITH12657 **(G–I)** at 7, 14 and 21 days after intravitreal injection of NMDA. The density of RGCs is represented with a color code ranging from purple or dark blue (0 RGCs/mm^2^) to red (≥2,500 RGCs/mm^2^).

In the control (intact right) retinas, the distribution of BRN3a^+^RGCs showed the typical higher densities in the upper retina along a strip located approximately 1 mm above the optic disc with maximum values in the superior-temporal quadrant, coinciding with the existence of the retinal visual streak (Nadal-Nicolas et al., 2009; Salinas-Navarro et al., 2009; Ortin-Martinez et al., 2010), a distribution that did not change with the time-intervals studied (Figures 9A–C). However, in the experimental (left) retinas, the distribution of RGCs, both in the vehicle- and in ITH12657-treated groups, was reduced at all times studied, in agreement with the above-shown quantitative results, and was diffuse throughout the retina (Figures 9D–I). Nevertheless, it was clear at all time intervals analyzed that the ITH12657-treated retinas showed higher Brn3a^+^RGCs densities at all time intervals analyzed when compared to vehicle-treated retinas (Figures 9G–I).

#### 3.2.4 *Ex vivo* analysis of the protection afforded by ITH12657 against NMDA excitotoxicity in the αRGC population and its subtypes

##### 3.2.4.1 Protection afforded by ITH12657 on αRGCs against NMDA excitotoxicity

**Figure 10:**
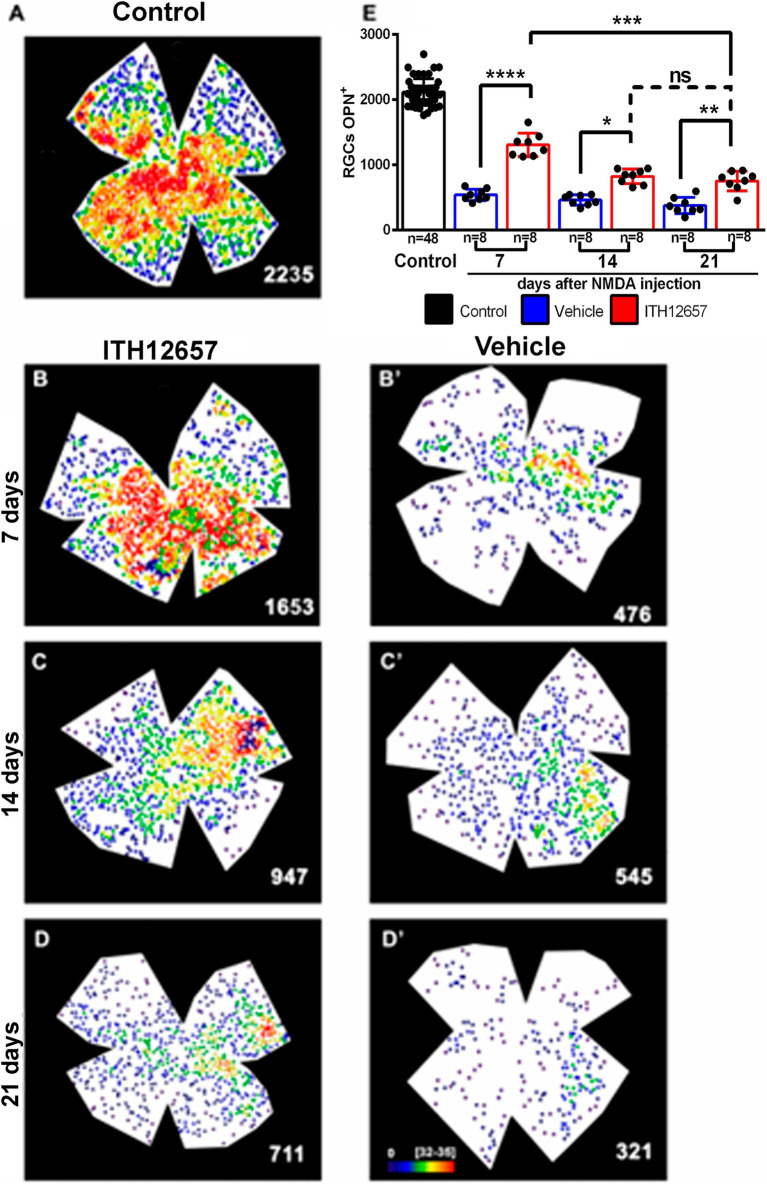
OPN^+^RGCs. Representative neighbor maps showing the topography of OPN^+^RGCs (αRGCs) in a control **(A)**, in ITH12657- **(B–D)** or in vehicle-treated **(B’–D’)** retinas. Neighbor maps illustrate the neuroprotective effects afforded by ITH12657 on the OPN^+^RGCs compared to vehicle at 7, 14 or 21 days. **(E)** Bar histogram showing mean total number of OPN^+^RGCs in control retinas (*n* = 48) and the protection or loss of OPN^+^RGC in the experimental retinas treated with ITH12657 (*n* = 8 per group) or vehicle (*n* = 8 per group). One-way ANOVA statistical significance difference ****p < 0.0001, ***p < 0.001, **p < 0.01; *p < 0.05; ns, not statistically difference *p* > 0.05. Neighbor maps color scale from 0 to 4 (purple) to ≥32–35 (red) neighbors.

The αRGCs were identified with OPN antibodies. In control retinas αRGCs showed the typical heterogeneous distribution within the retina with higher densities in the temporal hemiretina (Figure 10A) and mean total numbers of 2,135 ± 155 OPN^+^RGCs (αRGCs) (*n* = 48; Figure 10E), and these are in agreement with a recent report from our laboratory (Gallego-Ortega et al., 2022).

Seven days after intravitreal injection of NMDA, the experimental (left) retinas treated with vehicle showed a 26% survival of the αRGC population when compared with control (right intact) retinas (559 ± 90 OPN^+^RGCs 7d vehicle *vs* 2,135 ± 155 OPN^+^RGCs control), whereas the retinas treated with ITH12657 presented a 60% survival (1,294 ± 241 OPN^+^RGCs 7d ITH12657 vs. 2,135 ± 155 OPN^+^RGCs control) (Figure 10; Table 1).

When the vehicle-treated groups were compared at different survival intervals, there were no significant differences (One-way ANOVA, *p* > 0.05). That is, following intravitreal injection of NMDA there was a rapid and massive reduction of OPN^+^RGCs, already at 7 days, which did not progress further with time (559 ± 90 OPN^+^RGCs 7d vehicle vs. 729 ± 191 OPN^+^RGCs 21d vehicle). The groups treated with ITH12657 showed significant differences between 7 (60% survival) and 14 (39% survival) days (1,294 ± 241 OPN^+^RGCs 7d ITH12657 vs. 832 ± 133 OPN^+^RGCs 14d ITH12657, One-way ANOVA, *p* < 0.005), with no significant further reduction between 14 and 21 days, thus, suggesting that protection afforded by ITH12657 at 14 days was permanent (Figure 10).

##### 3.2.4.2 Protection afforded by ITH12657 against NMDA excitotoxicity on αONsRGCs

**Figure 11:**
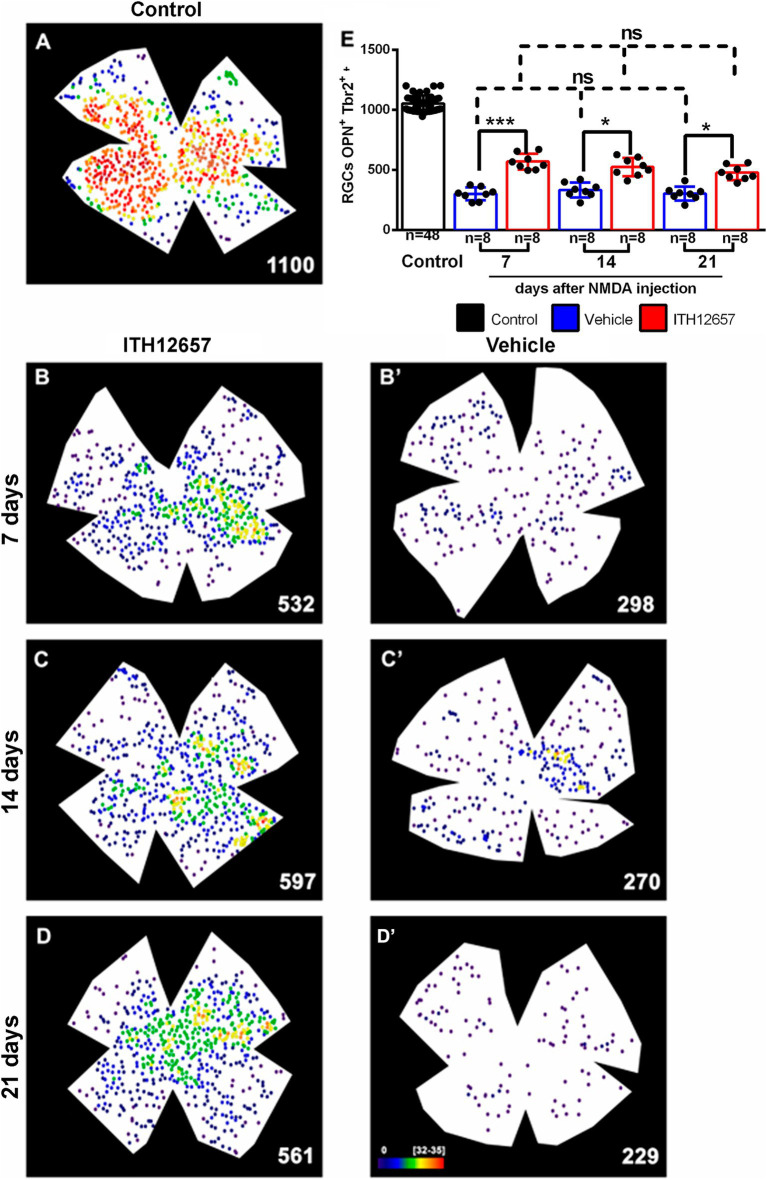
OPN^+^Tbr2^+^RGCs. Representative neighbor maps showing the topography of OPN^+^Tbr2^+^RGCs (αONsRGCs) in a control retina **(A)** and in ITH12657-treated **(B–D)** or Vehicle-treated **(B’–D’)** retinas. The neighbor maps illustrate the neuroprotective effects of ITH12657 on the OPN^+^Tbr2^+^RGCs compared to vehicle at 7, 14 or 21 days. **(E)** Bar histogram showing mean total numbers of OPN^+^Tbr2^+^RGCs in control retinas and the loss or neuroprotection of OPN^+^Tbr2^+^RGCs in the experimental retina treated with ITH12657 or vehicle. One-way ANOVA statistical significance difference ***p < 0.0001, *p < 0.05; ns, not statistically difference *p* > 0.05. Neighbor maps color scale from 0 to 4 (purple) to ≥32–35 (red) neighbors.

Cells doubly labeled with OPN and Tbr2 were dotted manually over retinal wholemounts. Such colocalization identifies α ON sustained RGCs (α-ONsRGCs) a subtype of αRGC that corresponds to the M4 type of the intrinsically photosensitive RGCs (Krieger et al., 2017; Gallego-Ortega et al., 2022). In control retinas these cells were distributed heterogeneously throughout the retina with the highest densities in the temporal hemiretina (Figure 11A) and a mean total number of 1,052 ± 55 OPN^+^Tbr2^+^RGCs, in agreement with recently published data (Gallego-Ortega et al., 2020, 2021; Figure 11).

The experimental retinas receiving intravitreal injection of NMDA and treatment with vehicle showed significant losses of α-ONsRGCs at 7 days of approximately 71% (One-way ANOVA, *p* < 0.005) when compared to the contralateral (intact right) group of retinas. These losses, which were evident on the topological maps did not present any particular pattern (Figure 11). The experimental groups of retinas treated with vehicle were compared with each other at different survival intervals, and there were no significant further losses of α-ONsRGCs (301 ± 27 OPN^+^Tbr2^+^RGC vehicle 7d vs. 295 ± 36 OPN^+^Tbr2^+^RGC vehicle 21d, One-way ANOVA, *p* > 0.05), indicating that NMDA-induced excitotoxicity resulted in a rapid and abrupt loss. Thus, the 71% loss that occurred at 7 days after NMDA was maintained until the last time interval studied of 21 days.

In contrast, the experimental retinas treated with ITH12657 showed at 7 days, a significantly greater survival of approximately 56% of the original OPN^+^Tbr2^+^RGC population, when compared to the vehicle-treated retinas (One-way ANOVA, *p* < 0.005), suggesting protection afforded by ITH12657 at 7 days. Such protection was permanent (Figure 11; Table 1) because there were no significant differences in total numbers of OPN^+^Tbr2^+^RGCs at 7, 14 or 21 days (569 ± 33 OPN^+^Tbr2^+^RGC 7d vs. 512 ± 53 OPN^+^Tbr2^+^RGC 14d vs. 479 ± 74 OPN^+^Tbr2^+^RGC 21d; One-way ANOVA, *p* > 0.05).

##### 3.2.4.3 Protection afforded by ITH12657 against NMDA excitotoxicity on αOFFRGCs

**Figure 12:**
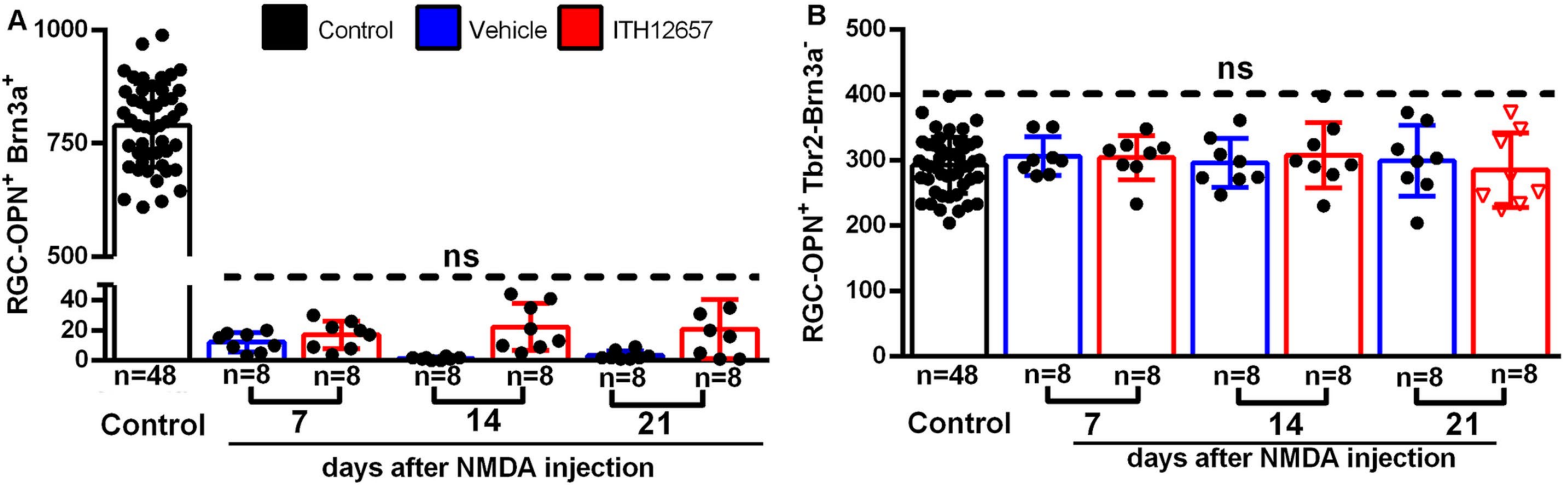
OPN^+^Brn3a^+^RGC and OPN^+^Tbr2^−^Brn3a^−^RGC populations. **(A)** Representative histogram of the OPN^+^Brn3a^+^RGC population (mean ± SD) comparing control eyes with experimental eyes NMDA-injected treated with vehicle (blue) or ITH12657 (red). **(B)** Representative histogram of the OPN^+^Tbr2^−^Brn3a^−^RGC population (mean ± SD) comparing control with experimental NMDA-injected eyes treated eyes with vehicle (blue) or ITH12657 (red). ns, not statistically difference One-way ANOVA, *p* > 0.05.

Cells doubly labeled with OPN and Brn3a were identified as α-OFFRGCs. In control retinas we found a mean total number of 811 ± 59 OPN^+^Brn3a^+^RGCs (Figure 12A). In experimental retinas, following NMDA-induced excitotoxicity and treatment with vehicle or ITH12657, we found great reductions to 1–5% of their original values by 7 days, with only 5–10 cells surviving in the retina. Thus, this type of α-RGC was extremely sensitive to NMDA-induced excitotoxicity and ITH12657 treatment did not afford protection of these RGCs (Figure 12A; Table 1).

##### 3.2.4.4 Protection afforded by ITH12657 against NMDA excitotoxicity on αONtRGCs

To investigate the responses of the α-ONtRGC population to NMDA-induced excitotoxicity and neuroprotection with ITH12657, we used OPN, Brn3a and Tbr2 antibodies. α-ONtRGCs cells are positive for OPN but negative for Brn3a and Tbr2. A total of 292 ± 12 cells were counted in control (right intact) retinas (Figure 12B; Table 1). In both the ITH12657- or vehicle-treated groups of experimental (left) retinas, there were no differences in total numbers of OPN^+^Brn3a^−^Tbr2^−^ RGCs with respect to control retinas (One-way ANOVA, *p* > 0.05), nor there were differences at survival intervals of 7, 14, or 21 days after NMDA-induced excitotoxicity (One-way ANOVA, *p* > 0.05) (Figure 12B; Table 1), indicating that the subtype of α-ONtRGCs (OPN^+^Brn3a^−^Tbr2^−^ RGCs) is fully resistant to NMDA-induced excitotoxicity.

## 4 Discussion

In this work, we use modern tools to identify, quantify and represent topologically the responses of different adult rat RGCs, the Brn3a^+^ and the αRGCs subtypes, to intravitreal injection of NMDA-induced excitotoxicity and systemic treatment with vehicle or ITH12657. We report for the first time that ITH12657 administered subcutaneously: (i) affords the best neuroprotective effects at a dose of 10 mg/kg, first administered 12 h prior to NMDA intravitreal injection and maintained for 3 days; (ii) prevents morphological and functional deficits of the retina; (iii) rescues significant proportions of Brn3a^+^RGCs up to 21 days; (iv) protects permanently a significant proportion of αONsRGCs; (v) does not alter the NMDA-induced massive loss of αOFFRGCs; and (vi) does not modify the resilience of αONtRGCs, which appear fully resistant to NMDA-induced excitotoxicity.

### 4.1 Characterization of the best dose, time-window, and treatment regime for ITH12657 afforded protection against NMDA-induced excitotoxicity

Our first group of experiments aimed at determining the best concentration, time-window, and administration frequency of ITH12567 administered subcutaneously to prevent NMDA-induced Brn3a^+^RGC loss. These studies showed that the best treatment regime for ITH 12657 was 10 mg/kg, starting 12 h before NMDA intravitreal injection and maintained for 3 additional days following NMDA IVI. This treatment protocol with ITH12657 resulted in substantial rescue of Brn3a^+^RGC (up to 72%) when compared to vehicle treatment (27%), and thus, such a treatment regime was implemented thereafter in the following experiments. It is well known that calcium channel blockade could influence retinal functionality and other pathways in a healthy animal (Boccuni and Fairless, 2022). It is possible that our best results obtained when treatment was started 12 h prior to NMDA administration, were due to the fact that the molecule has the necessary time to reach the retina before activation of the death pathways induced by NMDA excitotoxicity, which is very fast and aggressive (Vidal-Villegas et al., 2019). Other compounds like loncastuximab tesirine, thiamine, loncastuximab tesirine-lpyl, and thiamine mononitrate, are being investigated for their potential to prevent excessive calcium entry into neurons (Kopecky et al., 2014; Verma et al., 2022; Tsuruga et al., 2023). These compounds can protect neurons from damage, reduce oxidative stress and inflammation, and enhance mitochondrial function (Boccuni and Fairless, 2022). However, they may also impair synaptic transmission and plasticity, and affect neuronal development and differentiation (Boccuni and Fairless, 2022). The benefits and side effects of these compounds depend on their specific properties and the condition being treated.

### 4.2 *In vivo* protection afforded by ITH 12657 against NMDA-induced excitotoxicity

A second group of experiments further investigated the neuroprotective effects of ITH12657 on the retina. This was assessed *in vivo* morphologically with SD-OCT measurements of retinal layer thickness and functionally with full-field ERG recordings.

*In vivo* measurements with SD-OCT revealed a significant loss of total retinal thickness following NMDA-induced excitotoxicity and vehicle-treatment. These reductions may be explained on the basis of the rapid and massive loss of Brn3a^+^RGCs that follows NMDA-induced excitotoxicity, as shown previously (Vidal-Villegas et al., 2019) and confirmed in the present studies. Degeneration of this large population of RGCs involves the loss of neural processes that extend into the inner synaptic layer, resulting in retinal thickness reduction. In addition to RGCs, other neuronal types found in the inner retina and their resilience to excitotoxic damage may contribute to the inner retinal thinning. Indeed, other cell types present in this layer that have not been extensively studied include amacrine and bipolar cells. While amacrine cells have been shown to be sensitive to NMDA excitotoxicity in adult pigmented mice (Lam et al., 1999; Li et al., 1999; Akopian et al., 2014) and albino rats (Siliprandi et al., 1992; Huang et al., 2018), little is known about retinal bipolar cells. Retinal thinning following NMDA-induced excitotoxicity was significantly blunted with ITH12657 treatment. An effect that could be explained not only based on the protection exerted by ITH12657 on the Brn3a^+^RGC population but also over other retinal neurons that may be affected by NMDA excitotoxicity and were not studied histologically in the present experiments. Indeed, the ITH12657 treatment contributes to the maintenance of the b-wave amplitudes, which are produced mainly by retinal bipolar cells.

*In vivo* retinal functionality was assessed with full field electroretinograms following intravitreal injection of NMDA and treatment with vehicle or ITH12567. Our results show in the vehicle-treated group significant decreases in the mean amplitudes of the pSTR and b-wave of the mixed response. At 7 days, there was an 80% reduction in the mean amplitude of the positive component of the STR wave and a 68% reduction of the b-wave amplitude. NMDA-induced retinal excitotoxicity has been shown to induce the death of RGCs (Vidal-Villegas et al., 2019), amacrine cells (Milla-Navarro et al., 2021), and bipolar cells (Milla-Navarro et al., 2021), all of which express ionotropic glutamate receptors (Peng et al., 1995; Fletcher et al., 2000; Milla-Navarro et al., 2021). NMDA-induced excitotoxicity results in significant disorganization of the inner retina, which may also affect the outer retina. Our data show no alterations in the a-wave, which is primarily generated by photoreceptors, but this may be explained because secondary degeneration of the outer retina has been shown to occur for long periods after NMDA-induced excitotoxicity (Vidal-Villegas et al., 2019), and our present studies only extended up to 21 days post-injection.

Our *in vivo* results also demonstrate that following glutamatergic overstimulation there are progressive histological and functional changes, as reflected by retinal thinning and alterations of the main electrophysiological waves. Alterations in electroretinographic waves were observed as early as 7 days and persisted until 21 days post-injection, suggesting that NMDA-induced excitotoxicity produces immediate functional alterations in the retina, as shown anatomically in the present and previous studies (Vidal-Villegas et al., 2019). In contrast, animals treated systemically with ITH12657 did not exhibit such abrupt functional damage, and there was only a 40% reduction in pSTR and a 35% reduction in b-wave amplitude. Thus, our histological data on RGC survival and our electrophysiological data suggest that treatment with ITH12657 not only increases cell viability but also improves functionality following NMDA-induced excitotoxicity.

The progressive histological and functional changes following intravitreal injection of NMDA are consistent with previous studies from other research groups (Kermer et al., 2001; Hardingham et al., 2002; Gomez-Vicente et al., 2015; Huang et al., 2018; Wang et al., 2018) including our laboratory (Vidal-Villegas et al., 2019) that investigated RGC loss. At 7 days post-injection, there was a 74% reduction in the Brn3a^+^RGC population, with no subsequent reductions observed in animals that received only vehicle treatment. This loss is probably due to the activation of NMDA receptors, which trigger a massive influx of Ca^2+^ that acts as a second messenger to initiate apoptotic neuronal death pathways (Lebrun-Julien et al., 2009). However, the exact signaling pathways involved in NMDA-induced RGC death are not fully understood (Fahrenthold et al., 2018). ITH12657 significantly prevented the detrimental morphological and functional effects induced by NMDA intravitreal injection. It is tempting to suggest that ITH12657 may exert its protection by blocking voltage-dependent calcium channels, preventing excessive calcium influx and subsequent cell death via pathways activated by NMDA-induced excitotoxicity. Additionally, ITH12657 prevents inhibition of PP2A, an enzyme involved in Tau protein synthesis, which is essential for axonal maintenance and intracellular transport.

### 4.3 *Ex vivo* protection afforded by ITH 12657

In the second group of experiments, we further investigated *ex vivo* the neuroprotective effects of systemic administration of vehicle or the novel molecule ITH12657 on the retina against NMDA-induced excitotoxicity, and this was assessed with total counts of surviving Brn3a^+^RGCs, αRGCs and their subtypes (αONsRGCs; αONtRGCs and αOFFRGCs).

#### 4.3.1 Protection of Brn3a^+^RGCs afforded by ITH 12657

Intravitreal injection of NMDA resulted by 7 days in the loss of approximately 74% of the Br3a^+^RGC population without further loss, as previously reported (Vidal-Villegas et al., 2019). However, ITH12657 treatment resulted in significant protection with a survival of 58% of the Brn3a^+^RGC population at 14 days, which was permanent. It is tempting to suggest that the protection afforded by ITH12567 could be mediated by its capacity to block voltage-dependent calcium channels, thus preventing excessive calcium influx and subsequent cell death via pathways activated by NMDA injection. Moreover, ITH12657 prevents inhibition of PP2A, an enzyme involved in Tau protein synthesis, which is essential for axonal maintenance and intracellular transport (Gonzalez et al., 2018).

#### 4.3.2 Differential responses of αRGCs and their subtypes to NMDA-induced injury and protection with ITH 12657

In vehicle-treated groups of rats, following the NMDA IVI, the αRGC (OPN^+^RGC) population underwent a 73% loss at 7 days with no further loss during the rest of the study, whereas treatment with ITH12657 reduced this loss to 40% at 7 days and to 60% at 14 days, with such protection maintained for up to 21 days. The α-ONsRGC (OPN^+^Tbr2^+^RGC) population also exhibited different responses to NMDA-induced excitotoxicity and protection with ITH12657. The retinas receiving NMDA IVI and vehicle treatment, showed an approximately 28% survival of the original population of α-ONsRGC by 7 days, with no significant further losses with time. By contrast, the retinas treated with ITH12657 showed protection at 7 days that resulted in the survival of approximately 54% of the OPN^+^Tbr2^+^RGC population, with no significant variations at increasing survival intervals, suggesting that protection afforded by ITH12657 for OPN^+^Tbr2^+^RGCs was permanent (Figure 11). The α-OFF RGCs (OPN^+^Brn3a^+^RGCs) showed to be extremely sensitive to NMDA-induced excitotoxicity with only approximately 2% of the original population remaining by 7 days and no response to ITH12657 treatment (Figure 12A). The α-ONtRGCs (OPN^+^Tbr2^−^Brn3a^−^RGCs), on the contrary, were fully resistant to NMDA-induced excitotoxicity (Figure 12B), as has been previously documented for the melanopsin expressing RGCs M1-M3 (Vidal-Villegas et al., 2019).

Thus, it is interesting that αRGCs, a subclass that in rats comprises approximately 2.2% of all RGCs traced from the intra-orbital optic nerve (Nadal-Nicolas et al., 2015; Gallego-Ortega et al., 2021) and includes 4 of the 46 known types of RGCs (Rheaume et al., 2018; Tran et al., 2019; Goetz et al., 2022), exhibit a differential response to NMDA-induced excitotoxicity. At 7 days in animals that received vehicle treatment, the total population was reduced by 73, 72 or 98%, for the αRGC, the αONsRGC or the αOFFRGC populations, respectively, while the RGCα-ONt population was fully resistant to NMDA-induced excitotoxicity. It is interesting that αONsRGCs, which corresponds to M4, one type of ipRGCs (Schmidt et al., 2014; Krieger et al., 2017; Sonoda et al., 2020), does not show the typical resilience shown for other ipRGCs (M1-M3) to light induced phototoxicity (Garcia-Ayuso et al., 2017), acute ocular hypertension (Rovere et al., 2016), optic nerve injury (Nadal-Nicolas et al., 2015; Sanchez-Migallon et al., 2018; Gallego-Ortega et al., 2021; Vidal-Villegas et al., 2021a) or NMDA-induced excitotoxicity (Vidal-Villegas et al., 2019). Moreover, αONsRGCs have been shown to be particularly resistant to transient ocular hypertension (Zhao et al., 2023) perhaps due to their ability to modify their synaptic connections (El-Danaf and Huberman, 2015; Ou et al., 2016; Della Santina et al., 2021), but not to NMDA-induced excitotoxicity. Moreover, αRGC subtypes have been shown to exhibit different resilience to optic nerve injury, with the two sustained types being more resistant than the two transient types (Tran et al., 2019), while their resilience to NMDA-induced excitotoxicity differs based on their ON *vs* OFF responses, with a resilience for the ON- *vs* a great fragility for the OFF- αRGC subtypes. Thus, overall, our present results confirm the susceptibility of different RGCs to injury (Vidal-Villegas et al., 2019; Gallego-Ortega et al., 2021; Vidal-Villegas et al., 2021a) and add new data regarding the response of Brn3a^+^RGCs, αRGCs and their subtypes to NMDA-induced excitotoxicity and ITH12567 afforded protection.

An evolving concept over the last years relates to the idiosyncratic responses of RGCs to injury and protection (Vidal-Sanz et al., 2015a; Agudo-Barriuso et al., 2016). Indeed, it is currently thought that of the many different types of RGCs present in the rodent retina, a large number exhibit a differential response to injury and protection (Duan et al., 2015; Honda et al., 2019; Tran et al., 2019; Vidal-Villegas et al., 2019; Gallego-Ortega et al., 2021; Vidal-Villegas et al., 2021b; Zhao et al., 2023). Following optic nerve crush in mice, RNA sequencing revealed that not all RGC types respond similarly to injury (Tran et al., 2019). Our study supports these findings, as we report that different αRGC types exhibit varying responses to injury, with αONsRGC being more resistant, αOFFRGCs being extremely susceptible and αONtRGC being totally resistant. These results are consistent with other studies that have shown that the OFF pathway is more sensitive to NMDA excitotoxicity, with direction-selective RGC-ONs being more resistant than direction-selective RGC-OFFs (Milla-Navarro et al., 2021). Moreover, the response to ITH12657 afforded protection also varied greatly between αRGs and their subtypes, so that final survival at 21 days in animals that received ITH12657 treatment was 34, 45 or 2%, for the αRGC, the αONsRGC or the αOFFRGC populations, respectively, while the RGCα-ONt population was fully resistant to NMDA-induced excitotoxicity.

A previous study reported that αRGCs, immunodetected using an OPN antibody, are completely resistant and resilient to NMDA induced excitotoxicity (Christensen et al., 2019; Honda et al., 2019). Although αRGCs exhibit low expression of calcium-permeable glutamate receptors, high concentrations of NMDA can still induce death in these neurons. This may be the explanation for the differences between our results and those reported by Honda et al. (2019) and Honda et al. (2019) who used a 100-fold lower concentration of NMDA than we did. Additionally, their study was conducted on pigmented mouse retinas, and the susceptibility of cells in the albino rat retina may differ. Furthermore, their cell counting was performed by sampling retinal regions whereas we counted the total numbers of cells in retinal whole-mounts.

Treatment with the ITH12657 molecule had beneficial effects on neuroprotection of both the αRGC and Brn3a^+^RGC populations. Blockade of voltage-dependent calcium channels by ITH12657 may have prevented excessive calcium influx and subsequent cell death, resulting in increased survival of αRGCs and their subtype αONsRGCs in ITH12657-treated animals compared to those treated with vehicle alone. However, this effect was not observed in the αOFFRGC population. The high susceptibility of the αOFFRGC population to NMDA and the high concentration (100 mM) used in this study may have masked any neuroprotective effect of ITH12657. Further studies are needed to analyze if at lower concentrations of NMDA, ITH12657 would be neuroprotective on the αOFFRGC population.

In conclusion, following intravitreal injection of NMDA to induce excitotoxicity in the albino rat retina, systemic administration of ITH12657 (10 mg/kg) prevented significant retinal thinning, improved the functionality of RGCs and retinal interneurons, and protected Brn3a^+^RGCs, αRGCs and αONsRGCs populations, but not αOFFRGCs, while αONtRGCs appeared resistant to injury, further adding to previous findings about the idiosyncrasy of different RGCs to injury and protection (Vidal-Sanz et al., 2015a; Agudo-Barriuso et al., 2016; Vidal-Villegas et al., 2019, 2021a; Gallego-Ortega et al., 2021).

### 4.4 Limitations of the study

Neuroprotection studies in the retina need detailed knowledge about the response of different types of RGCs to injury and protection, as well as new strategies designed to impinge on various injury-activated death pathways.

It is tempting to suggest that the protective effects of ITH12657 are due to its capacity to block excess intracellular calcium influx. However, in this study the mechanism of action of ITH-12657 was not investigated, so additional experiments need to be designed to afford this purpose.

Our studies rely only in the immunohistochemical identification of RGCs by the expression or co-expression of specific markers, such as Brn3a, Osteopontin or Tbr2 to identify Brn3a + RGCs, αRGCs and their subtypes αONsRGCs, αONtRGCs and αOFFRGCs, respectively. But it is well known that NMDA-induced excitotoxicity can causes degeneration of other neuronal populations in the retina such us amacrine cells, and that injured neurons may modify the expression of typical markers (Vidal-Villegas et al., 2019). For this reason, a note of caution should be considered when interpreting the *ex vivo* and *in vivo* result.

## 5 Conclusion

We report for the first time the deleterious effects of intravitreal NMDA-induced excitotoxicity as well as the protective effects of subcutaneous administration of ITH12657, to blunt morphological and functional degeneration of the retina as well as the loss of Brn3a^+^RGCs, αRGCs and their subtype αONsRGCs. These results provide further evidence of the idiosyncratic responses to injury and protection of the main subtypes of a main class of retinal neurons, the αRGCs.

## Data availability statement

The raw data supporting the conclusions of this article will be made available by the authors, without undue reservation.

## Ethics statement

The animal study was approved by University of Murcia Ethical animal studies committee. The study was conducted in accordance with the local legislation and institutional requirements.

## Author contributions

JD: Conceptualization, Data curation, Formal analysis, Investigation, Methodology, Writing – original draft, Writing – review & editing. AG-O: Data curation, Formal analysis, Investigation, Methodology, Writing – original draft. MN-M: Data curation, Formal analysis, Investigation, Writing – original draft. BV-V: Data curation, Investigation, Writing – original draft. IB: Investigation, Resources, Writing – original draft. MB-R: Data curation, Writing – original draft. JB-G: Investigation, Writing – original draft. IF-B: Funding acquisition, Writing – review & editing. JP-J: Funding acquisition, Writing – review & editing. MV-P: Funding acquisition, Writing – review & editing. MA-T: Formal analysis, Funding acquisition, Supervision, Validation, Writing – review & editing. CdlR: Investigation, Methodology, Writing – review & editing. MV-S: Conceptualization, Data curation, Formal analysis, Funding acquisition, Investigation, Methodology, Supervision, Validation, Writing – original draft, Writing – review & editing.

## References

[ref1] Agudo-Barriuso M. Nadal-Nicolas F. M. Madeira M. H. Rovere G. Vidal-Villegas B. Vidal-Sanz M. (2016). Melanopsin expression is an indicator of the well-being of melanopsin-expressing retinal ganglion cells but not of their viability. Neural Regen. Res. 11, 1243–1244. doi: 10.4103/1673-5374.189182, PMID: 27651769 PMC5020820

[ref2] Akopian A. Atlasz T. Pan F. Wong S. Zhang Y. Volgyi B.. (2014). Gap junction-mediated death of retinal neurons is connexin and insult specific: a potential target for neuroprotection. J. Neurosci. 34, 10582–10591. doi: 10.1523/JNEUROSCI.1912-14.2014, PMID: 25100592 PMC4200109

[ref3] Alarcon-Martinez L. Aviles-Trigueros M. Galindo-Romero C. Valiente-Soriano J. Agudo-Barriuso M. Villa Pde L.. (2010). ERG changes in albino and pigmented mice after optic nerve transection. Vis. Res. 50, 2176–2187. doi: 10.1016/j.visres.2010.08.014, PMID: 20727908

[ref4] Alarcon-Martinez L. de la Villa P. Aviles-Trigueros M. Blanco R. Villegas-Perez M. P. Vidal-Sanz M. (2009). Short and long term axotomy-induced ERG changes in albino and pigmented rats. Mol. Vis. 15, 2373–2383. PMID: 19936311 PMC2779069

[ref5] Almasieh M. Wilson A. M. Morquette B. Cueva Vargas J. L. Di Polo A. (2012). The molecular basis of retinal ganglion cell death in glaucoma. Prog. Retin. Eye Res. 31, 152–181. doi: 10.1016/j.preteyeres.2011.11.002, PMID: 22155051

[ref6] Aviles-Trigueros M. Sauve Y. Lund R. D. Vidal-Sanz M. (2000). Selective innervation of retinorecipient brainstem nuclei by retinal ganglion cell axons regenerating through peripheral nerve grafts in adult rats. J. Neurosci. 20, 361–374. doi: 10.1523/JNEUROSCI.20-01-00361.2000, PMID: 10627613 PMC6774129

[ref7] Baden T. Berens P. Franke K. Roman Roson M. Bethge M. Euler T. (2016). The functional diversity of retinal ganglion cells in the mouse. Nature 529, 345–350. doi: 10.1038/nature16468, PMID: 26735013 PMC4724341

[ref8] Becker J. B. Prendergast B. J. Liang J. W. (2016). Female rats are not more variable than male rats: a meta-analysis of neuroscience studies. Biol. Sex Differ. 7:34. doi: 10.1186/s13293-016-0087-5, PMID: 27468347 PMC4962440

[ref9] Beery A. K. (2018). Inclusion of females does not increase variability in rodent research studies. Curr. Opin. Behav. Sci. 23, 143–149. doi: 10.1016/j.cobeha.2018.06.016, PMID: 30560152 PMC6294461

[ref11] Berson D. M. Castrucci A. M. Provencio I. (2010). Morphology and mosaics of melanopsin-expressing retinal ganglion cell types in mice. J. Comp. Neurol. 518, 2405–2422. doi: 10.1002/cne.22381, PMID: 20503419 PMC2895505

[ref12] Boccuni I. Fairless R. (2022). Retinal glutamate neurotransmission: from physiology to pathophysiological mechanisms of retinal ganglion cell degeneration. Life 12:638. doi: 10.3390/life12050638, PMID: 35629305 PMC9147752

[ref13] Chen H. Chen S. Zhang H. Wang S. Li Y. Meng X. (2022). N-methyl-D-aspartate receptor-mediated spinal cord ischemia-reperfusion injury and its protective mechanism. Folia Neuropathol. 60, 308–315. doi: 10.5114/fn.2022.118340, PMID: 36382483

[ref14] Choi D. W. (1988). Glutamate neurotoxicity and diseases of the nervous system. Neuron 1, 623–634. doi: 10.1016/0896-6273(88)90162-6 2908446

[ref15] Christensen I. Lu B. Yang N. Huang K. Wang P. Tian N. (2019). The susceptibility of retinal ganglion cells to glutamatergic excitotoxicity is type-specific. Front. Neurosci. 13:219. doi: 10.3389/fnins.2019.00219, PMID: 30930737 PMC6429039

[ref16] Della Santina L. Yu A. K. Harris S. C. Solino M. Garcia Ruiz T. Most J.. (2021). Disassembly and rewiring of a mature converging excitatory circuit following injury. Cell Rep. 36:109463. doi: 10.1016/j.celrep.2021.109463, PMID: 34348156 PMC8381591

[ref17] DeParis S. Caprara C. Grimm C. (2012). Intrinsically photosensitive retinal ganglion cells are resistant to N-methyl-D-aspartic acid excitotoxicity. Mol. Vis. 18, 2814–2827. PMID: 23233784 PMC3519378

[ref18] Di Pierdomenico J. Gallego-Ortega A. Martinez-Vacas A. Garcia-Bernal D. Vidal-Sanz M. Villegas-Perez M. P.. (2022a). Intravitreal and subretinal syngeneic bone marrow mononuclear stem cell transplantation improves photoreceptor survival but does not ameliorate retinal function in two rat models of retinal degeneration. Acta Ophthalmol. 100, e1313–e1331. doi: 10.1111/aos.15165, PMID: 35514078

[ref19] Di Pierdomenico J. Henderson D. C. M. Giammaria S. Smith V. L. Jamet A. J. Smith C. A.. (2022b). Age and intraocular pressure in murine experimental glaucoma. Prog. Retin. Eye Res. 88:101021. doi: 10.1016/j.preteyeres.2021.101021, PMID: 34801667

[ref20] Di Pierdomenico J. Muñoz M. N. Ortega A. G. Ruiz M. B. Garro J. M. B. Perez M. P. V.. (2022c). Neuroprotective effects of ITH-IB6 against excitotoxicity-induced retinal injury. Acta Ophthalmol 100. doi: 10.1111/j.1755-3768.2022.0243

[ref21] Dreyer E. B. Zurakowski D. Schumer R. A. Podos S. M. Lipton S. A. (1996). Elevated glutamate levels in the vitreous body of humans and monkeys with glaucoma. Arch. Ophthalmol. 114, 299–305. doi: 10.1001/archopht.1996.01100130295012, PMID: 8600890

[ref22] Duan X. Qiao M. Bei F. Kim I. J. He Z. Sanes J. R. (2015). Subtype-specific regeneration of retinal ganglion cells following axotomy: effects of osteopontin and mTOR signaling. Neuron 85, 1244–1256. doi: 10.1016/j.neuron.2015.02.017, PMID: 25754821 PMC4391013

[ref23] El-Danaf R. N. Huberman A. D. (2015). Characteristic patterns of dendritic remodeling in early-stage glaucoma: evidence from genetically identified retinal ganglion cell types. J. Neurosci. 35, 2329–2343. doi: 10.1523/JNEUROSCI.1419-14.2015, PMID: 25673829 PMC6605614

[ref24] Estevez M. E. Fogerson P. M. Ilardi M. C. Borghuis B. G. Chan E. Weng S.. (2012). Form and function of the M4 cell, an intrinsically photosensitive retinal ganglion cell type contributing to geniculocortical vision. J. Neurosci. 32, 13608–13620. doi: 10.1523/JNEUROSCI.1422-12.2012, PMID: 23015450 PMC3474539

[ref25] Evangeliou E. Plemmenos G. Chalazias A. Piperi C. (2023). Impact of TRP channels in Oral pathology and therapeutic targeting options: a narrative review. Curr. Top. Med. Chem. 23, 1559–1573. doi: 10.2174/1568026623666230331110443, PMID: 36999699

[ref26] Fahrenthold B. K. Fernandes K. A. Libby R. T. (2018). Assessment of intrinsic and extrinsic signaling pathway in excitotoxic retinal ganglion cell death. Sci. Rep. 8:4641. doi: 10.1038/s41598-018-22848-y, PMID: 29545615 PMC5854579

[ref27] Fletcher E. L. Hack I. Brandstatter J. H. Wassle H. (2000). Synaptic localization of NMDA receptor subunits in the rat retina. J. Comp. Neurol. 420, 98–112. doi: 10.1002/(SICI)1096-9861(20000424)420:1<98::AID-CNE7>3.0.CO;2-U, PMID: 10745222

[ref28] Galindo-Romero C. Jimenez-Lopez M. Garcia-Ayuso D. Salinas-Navarro M. Nadal-Nicolas F. M. Agudo-Barriuso M.. (2013). Number and spatial distribution of intrinsically photosensitive retinal ganglion cells in the adult albino rat. Exp. Eye Res. 108, 84–93. doi: 10.1016/j.exer.2012.12.010, PMID: 23295345

[ref29] Gallego-Ortega A. Norte-Munoz M. Di Pierdomenico J. Aviles-Trigueros M. de la Villa P. Valiente-Soriano F. J.. (2022). Alpha retinal ganglion cells in pigmented mice retina: number and distribution. Front. Neuroanat. 16:1054849. doi: 10.3389/fnana.2022.1054849, PMID: 36530520 PMC9751430

[ref30] Gallego-Ortega A. Norte-Munoz M. Miralles de Imperial-Ollero J. A. Bernal-Garro J. M. Valiente-Soriano F. J. de la Villa Polo P.. (2020). Functional and morphological alterations in a glaucoma model of acute ocular hypertension. Prog. Brain Res. 256, 1–29. doi: 10.1016/bs.pbr.2020.07.003 32958209

[ref31] Gallego-Ortega A. Vidal-Villegas B. Norte-Munoz M. Salinas-Navarro M. Aviles-Trigueros M. Villegas-Perez M. P.. (2021). 7,8-Dihydroxiflavone maintains retinal functionality and protects various types of RGCs in adult rats with optic nerve transection. Int. J. Mol. Sci. 22:11815. doi: 10.3390/ijms222111815, PMID: 34769247 PMC8584116

[ref32] Garcia-Ayuso D. Galindo-Romero C. Di Pierdomenico J. Vidal-Sanz M. Agudo-Barriuso M. Villegas Perez M. P. (2017). Light-induced retinal degeneration causes a transient downregulation of melanopsin in the rat retina. Exp. Eye Res. 161, 10–16. doi: 10.1016/j.exer.2017.05.010, PMID: 28552384

[ref33] Garcia-Layana A. Figueroa M. S. Arias L. Araiz J. Ruiz-Moreno J. M. Garcia-Arumi J.. (2015). Individualized therapy with ranibizumab in wet age-related macular degeneration. J. Ophthalmol. 2015:412903. doi: 10.1155/2015/412903, PMID: 26491550 PMC4600506

[ref34] Goetz J. Jessen Z. F. Jacobi A. Mani A. Cooler S. Greer D.. (2022). Unified classification of mouse retinal ganglion cells using function, morphology, and gene expression. Cell Rep. 40:111040. doi: 10.1016/j.celrep.2022.111040, PMID: 35830791 PMC9364428

[ref35] Gomez-Vicente V. Lax P. Fernandez-Sanchez L. Rondon N. Esquiva G. Germain F.. (2015). Neuroprotective effect of tauroursodeoxycholic acid on N-methyl-D-aspartate-induced retinal ganglion cell degeneration. PLoS One 10:e0137826. doi: 10.1371/journal.pone.0137826, PMID: 26379056 PMC4574963

[ref36] Gonzalez D. Arribas R. L. Viejo L. Lajarin-Cuesta R. de Los Rios C. (2018). Substituent effect of N-benzylated gramine derivatives that prevent the PP2A inhibition and dissipate the neuronal Ca(2+) overload, as a multitarget strategy for the treatment of Alzheimer’s disease. Bioorg. Med. Chem. 26, 2551–2560. doi: 10.1016/j.bmc.2018.04.019, PMID: 29656989

[ref37] Hahn J. Monavarfeshani A. Qiao M. Kao A. H. Kolsch Y. Kumar A.. (2023). Evolution of neuronal cell classes and types in the vertebrate retina. Nature 624, 415–424. doi: 10.1038/s41586-023-06638-9, PMID: 38092908 PMC10719112

[ref38] Hardingham G. E. Fukunaga Y. Bading H. (2002). Extrasynaptic NMDARs oppose synaptic NMDARs by triggering CREB shut-off and cell death pathways. Nat. Neurosci. 5, 405–414. doi: 10.1038/nn835, PMID: 11953750

[ref39] Honda S. Namekata K. Kimura A. Guo X. Harada C. Murakami A.. (2019). Survival of alpha and intrinsically photosensitive retinal ganglion cells in NMDA-induced neurotoxicity and a mouse model of Normal tension Glaucoma. Invest. Ophthalmol. Vis. Sci. 60, 3696–3707. doi: 10.1167/iovs.19-27145 31487370

[ref40] Huang W. Hu F. Wang M. Gao F. Xu P. Xing C.. (2018). Comparative analysis of retinal ganglion cell damage in three glaucomatous rat models. Exp. Eye Res. 172, 112–122. doi: 10.1016/j.exer.2018.03.019, PMID: 29605491

[ref41] Huang W. Xu Q. Su J. Tang L. Hao Z. Z. Xu C.. (2022). Linking transcriptomes with morphological and functional phenotypes in mammalian retinal ganglion cells. Cell Rep. 40:111322. doi: 10.1016/j.celrep.2022.111322, PMID: 36103830

[ref42] Ito A. Tsuda S. Kunikata H. Toshifumi A. Sato K. Nakazawa T. (2019). Assessing retinal ganglion cell death and neuroprotective agents using real time imaging. Brain Res. 1714, 65–72. doi: 10.1016/j.brainres.2019.02.008, PMID: 30753816

[ref43] Izzotti A. Bagnis A. Sacca S. C. (2006). The role of oxidative stress in glaucoma. Mutat. Res. 612, 105–114. doi: 10.1016/j.mrrev.2005.11.001 16413223

[ref44] Kermer P. Klocker N. Bahr M. (2001). Modulation of metabotropic glutamate receptors fails to prevent the loss of adult rat retinal ganglion cells following axotomy or N-methyl-D-aspartate lesion in vivo. Neurosci. Lett. 315, 117–120. doi: 10.1016/s0304-3940(01)02318-7 11716977

[ref45] Kobayashi M. Hirooka K. Ono A. Nakano Y. Nishiyama A. Tsujikawa A. (2017). The relationship between the renin-angiotensin-aldosterone system and NMDA receptor-mediated signal and the prevention of retinal ganglion cell death. Invest. Ophthalmol. Vis. Sci. 58, 1397–1403. doi: 10.1167/iovs.16-21001, PMID: 28253402

[ref46] Kopecky B. J. Liang R. Bao J. (2014). T-type calcium channel blockers as neuroprotective agents. Pflugers Arch. 466, 757–765. doi: 10.1007/s00424-014-1454-x, PMID: 24563219 PMC4005039

[ref47] Krieger B. Qiao M. Rousso D. L. Sanes J. R. Meister M. (2017). Four alpha ganglion cell types in mouse retina: function, structure, and molecular signatures. PLoS One 12:e0180091. doi: 10.1371/journal.pone.0180091, PMID: 28753612 PMC5533432

[ref48] Lajarin-Cuesta R. Nanclares C. Arranz-Tagarro J. A. Gonzalez-Lafuente L. Arribas R. L. Araujo de Brito M.. (2016). Gramine derivatives targeting ca(2+) channels and Ser/Thr phosphatases: a new dual strategy for the treatment of neurodegenerative diseases. J. Med. Chem. 59, 6265–6280. doi: 10.1021/acs.jmedchem.6b00478, PMID: 27280380

[ref49] Lam T. T. Abler A. S. Kwong J. M. Tso M. O. (1999). N-methyl-D-aspartate (NMDA)--induced apoptosis in rat retina. Invest. Ophthalmol. Vis. Sci. 40, 2391–2397. PMID: 10476807

[ref50] Lam T. T. Siew E. Chu R. Tso M. O. (1997). Ameliorative effect of MK-801 on retinal ischemia. J. Ocul. Pharmacol. Ther. 13, 129–137. doi: 10.1089/jop.1997.13.129, PMID: 9090613

[ref51] Lambuk L. Iezhitsa I. Agarwal R. Bakar N. S. Agarwal P. Ismail N. M. (2019). Antiapoptotic effect of taurine against NMDA-induced retinal excitotoxicity in rats. Neurotoxicology 70, 62–71. doi: 10.1016/j.neuro.2018.10.009, PMID: 30385388

[ref52] Lebrun-Julien F. Duplan L. Pernet V. Osswald I. Sapieha P. Bourgeois P.. (2009). Excitotoxic death of retinal neurons in vivo occurs via a non-cell-autonomous mechanism. J. Neurosci. 29, 5536–5545. doi: 10.1523/JNEUROSCI.0831-09.2009, PMID: 19403821 PMC6665839

[ref53] Li Y. Schlamp C. L. Nickells R. W. (1999). Experimental induction of retinal ganglion cell death in adult mice. Invest. Ophthalmol. Vis. Sci. 40, 1004–1008. PMID: 10102300

[ref54] Li S. Yang C. Zhang L. Gao X. Wang X. Liu W.. (2016). Promoting axon regeneration in the adult CNS by modulation of the melanopsin/GPCR signaling. Proc. Natl. Acad. Sci. U. S. A. 113, 1937–1942. doi: 10.1073/pnas.1523645113, PMID: 26831088 PMC4763730

[ref55] Lindqvist N. Peinado-Ramonn P. Vidal-Sanz M. Hallbook F. (2004). GDNF, ret, GFRalpha1 and 2 in the adult rat retino-tectal system after optic nerve transection. Exp. Neurol. 187, 487–499. doi: 10.1016/j.expneurol.2004.02.002, PMID: 15144875

[ref56] Lucas D. R. Newhouse J. P. (1957). The toxic effect of sodium L-glutamate on the inner layers of the retina. A.M.A. Arch. Ophthalmol. 58, 193–201. doi: 10.1001/archopht.1957.00940010205006 13443577

[ref57] Manabe S. Gu Z. Lipton S. A. (2005). Activation of matrix metalloproteinase-9 via neuronal nitric oxide synthase contributes to NMDA-induced retinal ganglion cell death. Invest. Ophthalmol. Vis. Sci. 46, 4747–4753. doi: 10.1167/iovs.05-0128, PMID: 16303975

[ref58] Manev H. Favaron M. Guidotti A. Costa E. (1989). Delayed increase of Ca2+ influx elicited by glutamate: role in neuronal death. Mol. Pharmacol. 36, 106–112. PMID: 2568579

[ref59] McKerracher L. Vidal-Sanz M. Aguayo A. J. (1990a). Slow transport rates of cytoskeletal proteins change during regeneration of axotomized retinal neurons in adult rats. J. Neurosci. 10, 641–648. doi: 10.1523/JNEUROSCI.10-02-00641.1990, PMID: 2106015 PMC6570155

[ref60] McKerracher L. Vidal-Sanz M. Essagian C. Aguayo A. J. (1990b). Selective impairment of slow axonal transport after optic nerve injury in adult rats. J. Neurosci. 10, 2834–2841. doi: 10.1523/JNEUROSCI.10-08-02834.1990, PMID: 1696983 PMC6570264

[ref61] Milla-Navarro S. Diaz-Tahoces A. Ortuno-Lizaran I. Fernandez E. Cuenca N. Germain F.. (2021). Visual Disfunction due to the selective effect of glutamate agonists on retinal cells. Int. J. Mol. Sci. 22:6245. doi: 10.3390/ijms22126245, PMID: 34200611 PMC8230349

[ref62] Nadal-Nicolas F. M. Galindo-Romero C. Lucas-Ruiz F. Marsh-Amstrong N. Li W. Vidal-Sanz M.. (2023). Pan-retinal ganglion cell markers in mice, rats, and rhesus macaques. Zool. Res. 44, 1–23. doi: 10.24272/j.issn.2095-8137.2022.308, PMID: 36594396 PMC9841181

[ref63] Nadal-Nicolas F. M. Jimenez-Lopez M. Salinas-Navarro M. Sobrado-Calvo P. Alburquerque-Bejar J. J. Vidal-Sanz M.. (2012). Whole number, distribution and co-expression of brn3 transcription factors in retinal ganglion cells of adult albino and pigmented rats. PLoS One 7:e49830. doi: 10.1371/journal.pone.0049830, PMID: 23166779 PMC3500320

[ref64] Nadal-Nicolas F. M. Jimenez-Lopez M. Sobrado-Calvo P. Nieto-Lopez L. Canovas-Martinez I. Salinas-Navarro M.. (2009). Brn3a as a marker of retinal ganglion cells: qualitative and quantitative time course studies in naive and optic nerve-injured retinas. Invest. Ophthalmol. Vis. Sci. 50, 3860–3868. doi: 10.1167/iovs.08-3267, PMID: 19264888

[ref65] Nadal-Nicolas F. M. Salinas-Navarro M. Jimenez-Lopez M. Sobrado-Calvo P. Villegas-Perez M. P. Vidal-Sanz M.. (2014). Displaced retinal ganglion cells in albino and pigmented rats. Front. Neuroanat. 8:99. doi: 10.3389/fnana.2014.00099 25339868 PMC4186482

[ref66] Nadal-Nicolas F. M. Sobrado-Calvo P. Jimenez-Lopez M. Vidal-Sanz M. Agudo-Barriuso M. (2015). Long-term effect of optic nerve axotomy on the retinal ganglion cell layer. Invest. Ophthalmol. Vis. Sci. 56, 6095–6112. doi: 10.1167/iovs.15-17195, PMID: 26393669

[ref67] Nadal-Nicolas F. M. Vidal-Sanz M. Agudo-Barriuso M. (2018). The aging rat retina: from function to anatomy. Neurobiol. Aging 61, 146–168. doi: 10.1016/j.neurobiolaging.2017.09.021 29080498

[ref68] Nassi J. J. Callaway E. M. (2009). Parallel processing strategies of the primate visual system. Nat. Rev. Neurosci. 10, 360–372. doi: 10.1038/nrn2619, PMID: 19352403 PMC2771435

[ref69] Ortin-Martinez A. Jimenez-Lopez M. Nadal-Nicolas F. M. Salinas-Navarro M. Alarcon-Martinez L. Sauve Y.. (2010). Automated quantification and topographical distribution of the whole population of S- and L-cones in adult albino and pigmented rats. Invest. Ophthalmol. Vis. Sci. 51, 3171–3183. doi: 10.1167/iovs.09-4861, PMID: 20071667

[ref70] Ortin-Martinez A. Salinas-Navarro M. Nadal-Nicolas F. M. Jimenez-Lopez M. Valiente-Soriano F. J. Garcia-Ayuso D.. (2015). Laser-induced ocular hypertension in adult rats does not affect non-RGC neurons in the ganglion cell layer but results in protracted severe loss of cone-photoreceptors. Exp. Eye Res. 132, 17–33. doi: 10.1016/j.exer.2015.01.006 25576772

[ref71] Ortin-Martinez A. Valiente-Soriano F. J. Garcia-Ayuso D. Alarcon-Martinez L. Jimenez-Lopez M. Bernal-Garro J. M.. (2014). A novel in vivo model of focal light emitting diode-induced cone-photoreceptor phototoxicity: neuroprotection afforded by brimonidine, BDNF, PEDF or bFGF. PLoS One 9:e113798. doi: 10.1371/journal.pone.0113798, PMID: 25464513 PMC4252057

[ref72] Ou Y. Jo R. E. Ullian E. M. Wong R. O. Della Santina L. (2016). Selective vulnerability of specific retinal ganglion cell types and synapses after transient ocular hypertension. J. Neurosci. 36, 9240–9252. doi: 10.1523/JNEUROSCI.0940-16.2016, PMID: 27581463 PMC5005727

[ref73] Peng Y. W. Blackstone C. D. Huganir R. L. Yau K. W. (1995). Distribution of glutamate receptor subtypes in the vertebrate retina. Neuroscience 66, 483–497. doi: 10.1016/0306-4522(94)00569-q 7477889

[ref74] Pichavaram P. Palani C. D. Patel C. Xu Z. Shosha E. Fouda A. Y.. (2018). Targeting polyamine oxidase to prevent excitotoxicity-induced retinal neurodegeneration. Front. Neurosci. 12:956. doi: 10.3389/fnins.2018.00956, PMID: 30686964 PMC6335392

[ref75] Quattrochi L. E. Stabio M. E. Kim I. Ilardi M. C. Michelle Fogerson P. Leyrer M. L.. (2019). The M6 cell: a small-field bistratified photosensitive retinal ganglion cell. J. Comp. Neurol. 527, 297–311. doi: 10.1002/cne.24556, PMID: 30311650 PMC6594700

[ref76] Rheaume B. A. Jereen A. Bolisetty M. Sajid M. S. Yang Y. Renna K.. (2018). Single cell transcriptome profiling of retinal ganglion cells identifies cellular subtypes. Nat. Commun. 9:2759. doi: 10.1038/s41467-018-05134-3, PMID: 30018341 PMC6050223

[ref77] Rovere G. Nadal-Nicolas F. M. Agudo-Barriuso M. Sobrado-Calvo P. Nieto-Lopez L. Nucci C.. (2015). Comparison of retinal nerve fiber layer thinning and retinal ganglion cell loss after optic nerve transection in adult albino rats. Invest. Ophthalmol. Vis. Sci. 56, 4487–4498. doi: 10.1167/iovs.15-17145, PMID: 26193926

[ref78] Rovere G. Nadal-Nicolas F. M. Wang J. Bernal-Garro J. M. Garcia-Carrillo N. Villegas-Perez M. P.. (2016). Melanopsin-containing or non-melanopsin-containing retinal ganglion cells response to acute ocular hypertension with or without brain-derived neurotrophic factor neuroprotection. Invest. Ophthalmol. Vis. Sci. 57, 6652–6661. doi: 10.1167/iovs.16-20146 27930778

[ref79] Salinas-Navarro M. Mayor-Torroglosa S. Jimenez-Lopez M. Aviles-Trigueros M. Holmes T. M. Lund R. D.. (2009). A computerized analysis of the entire retinal ganglion cell population and its spatial distribution in adult rats. Vis. Res. 49, 115–126. doi: 10.1016/j.visres.2008.09.029, PMID: 18952118

[ref80] Sanchez-Migallon M. C. Valiente-Soriano F. J. Nadal-Nicolas F. M. Di Pierdomenico J. Vidal-Sanz M. Agudo-Barriuso M. (2018). Survival of melanopsin expressing retinal ganglion cells long term after optic nerve trauma in mice. Exp. Eye Res. 174, 93–97. doi: 10.1016/j.exer.2018.05.029 29856984

[ref81] Sanes J. R. Masland R. H. (2015). The types of retinal ganglion cells: current status and implications for neuronal classification. Annu. Rev. Neurosci. 38, 221–246. doi: 10.1146/annurev-neuro-071714-034120, PMID: 25897874

[ref82] Schmidt T. M. Alam N. M. Chen S. Kofuji P. Li W. Prusky G. T.. (2014). A role for melanopsin in alpha retinal ganglion cells and contrast detection. Neuron 82, 781–788. doi: 10.1016/j.neuron.2014.03.022, PMID: 24853938 PMC4083763

[ref83] Schmidt T. M. Chen S. K. Hattar S. (2011a). Intrinsically photosensitive retinal ganglion cells: many subtypes, diverse functions. Trends Neurosci. 34, 572–580. doi: 10.1016/j.tins.2011.07.001, PMID: 21816493 PMC3200463

[ref84] Schmidt T. M. Do M. T. Dacey D. Lucas R. Hattar S. Matynia A. (2011b). Melanopsin-positive intrinsically photosensitive retinal ganglion cells: from form to function. J. Neurosci. 31, 16094–16101. doi: 10.1523/JNEUROSCI.4132-11.2011, PMID: 22072661 PMC3267581

[ref85] Schuettauf F. Naskar R. Vorwerk C. K. Zurakowski D. Dreyer E. B. (2000). Ganglion cell loss after optic nerve crush mediated through AMPA-kainate and NMDA receptors. Invest. Ophthalmol. Vis. Sci. 41, 4313–4316. PMID: 11095632

[ref86] Siliprandi R. Canella R. Carmignoto G. Schiavo N. Zanellato A. Zanoni R.. (1992). N-methyl-D-aspartate-induced neurotoxicity in the adult rat retina. Vis. Neurosci. 8, 567–573. doi: 10.1017/s0952523800005666, PMID: 1586655

[ref87] Sonoda T. Okabe Y. Schmidt T. M. (2020). Overlapping morphological and functional properties between M4 and M5 intrinsically photosensitive retinal ganglion cells. J. Comp. Neurol. 528, 1028–1040. doi: 10.1002/cne.24806, PMID: 31691279 PMC7007370

[ref88] Stavrovskaya I. G. Kristal B. S. (2005). The powerhouse takes control of the cell: is the mitochondrial permeability transition a viable therapeutic target against neuronal dysfunction and death? Free Radic. Biol. Med. 38, 687–697. doi: 10.1016/j.freeradbiomed.2004.11.032, PMID: 15721979

[ref89] Sweeney N. T. James K. N. Nistorica A. Lorig-Roach R. M. Feldheim D. A. (2019). Expression of transcription factors divides retinal ganglion cells into distinct classes. J. Comp. Neurol. 527, 225–235. doi: 10.1002/cne.24172, PMID: 28078709 PMC9444162

[ref90] Tezel G. (2013). Immune regulation toward immunomodulation for neuroprotection in glaucoma. Curr. Opin. Pharmacol. 13, 23–31. doi: 10.1016/j.coph.2012.09.013, PMID: 23084793 PMC3529855

[ref91] Tran N. M. Shekhar K. Whitney I. E. Jacobi A. Benhar I. Hong G.. (2019). Single-cell profiles of retinal ganglion cells differing in resilience to injury reveal neuroprotective genes. Neuron 104, 1039–1055.e12. doi: 10.1016/j.neuron.2019.11.006, PMID: 31784286 PMC6923571

[ref92] Tsuruga H. Murata H. Araie M. Aihara M. (2023). Neuroprotective effect of the calcium channel blocker nilvadipine on retinal ganglion cell death in a mouse ocular hypertension model. Heliyon 9:e13812. doi: 10.1016/j.heliyon.2023.e13812, PMID: 36879972 PMC9984798

[ref93] Valiente-Soriano F. J. Garcia-Ayuso D. Ortin-Martinez A. Jimenez-Lopez M. Galindo-Romero C. Villegas-Perez M. P.. (2014). Distribution of melanopsin positive neurons in pigmented and albino mice: evidence for melanopsin interneurons in the mouse retina. Front. Neuroanat. 8:131. doi: 10.3389/fnana.2014.00131, PMID: 25477787 PMC4238377

[ref94] Valiente-Soriano F. J. Nadal-Nicolas F. M. Salinas-Navarro M. Jimenez-Lopez M. Bernal-Garro J. M. Villegas-Perez M. P.. (2015). BDNF rescues RGCs but not intrinsically photosensitive RGCs in ocular hypertensive albino rat retinas. Invest. Ophthalmol. Vis. Sci. 56, 1924–1936. doi: 10.1167/iovs.15-16454, PMID: 25722208

[ref95] Verma M. Lizama B. N. Chu C. T. (2022). Excitotoxicity, calcium and mitochondria: a triad in synaptic neurodegeneration. Transl. Neurodegener. 11:3. doi: 10.1186/s40035-021-00278-7, PMID: 35078537 PMC8788129

[ref96] Vidal-Sanz M. Aviles-Trigueros M. Whiteley S. J. Sauve Y. Lund R. D. (2002). Reinnervation of the pretectum in adult rats by regenerated retinal ganglion cell axons: anatomical and functional studies. Prog. Brain Res. 137, 443–452. doi: 10.1016/s0079-6123(02)37035-3 12440386

[ref97] Vidal-Sanz M. Galindo-Romero C. Valiente-Soriano F. J. Nadal-Nicolas F. M. Ortin-Martinez A. Rovere G.. (2017). Shared and differential retinal responses against optic nerve injury and ocular hypertension. Front. Neurosci. 11:235. doi: 10.3389/fnins.2017.00235, PMID: 28491019 PMC5405145

[ref98] Vidal-Sanz M. Nadal-Nicolas F. M. Valiente-Soriano F. J. Agudo-Barriuso M. Villegas-Perez M. P. (2015a). Identifying specific RGC types may shed light on their idiosyncratic responses to neuroprotection. Neural Regen. Res. 10, 1228–1230. doi: 10.4103/1673-5374.162751, PMID: 26487846 PMC4590231

[ref99] Vidal-Sanz M. Salinas-Navarro M. Nadal-Nicolas F. M. Alarcon-Martinez L. Valiente-Soriano F. J. de Imperial J. M.. (2012). Understanding glaucomatous damage: anatomical and functional data from ocular hypertensive rodent retinas. Prog. Retin. Eye Res. 31, 1–27. doi: 10.1016/j.preteyeres.2011.08.001, PMID: 21946033

[ref100] Vidal-Sanz M. Valiente-Soriano F. J. Ortin-Martinez A. Nadal-Nicolas F. M. Jimenez-Lopez M. Salinas-Navarro M.. (2015b). Retinal neurodegeneration in experimental glaucoma. Prog. Brain Res. 220, 1–35. doi: 10.1016/bs.pbr.2015.04.008 26497783

[ref101] Vidal-Villegas B. Di Pierdomenico J. Gallego-Ortega A. Galindo-Romero C. Martinez-de-la-Casa J. M. Garcia-Feijoo J.. (2021a). Systemic treatment with 7,8-Dihydroxiflavone activates TtkB and affords protection of two different retinal ganglion cell populations against axotomy in adult rats. Exp. Eye Res. 210:108694. doi: 10.1016/j.exer.2021.108694, PMID: 34245756

[ref102] Vidal-Villegas B. Di Pierdomenico J. Miralles de Imperial-Ollero J. A. Ortin-Martinez A. Nadal-Nicolas F. M. Bernal-Garro J. M.. (2019). Melanopsin(+)RGCs are fully resistant to NMDA-induced excitotoxicity. Int. J. Mol. Sci. 20:3012. doi: 10.3390/ijms20123012, PMID: 31226772 PMC6627747

[ref103] Vidal-Villegas B. Gallego-Ortega A. Miralles de Imperial-Ollero J. A. Martinez de la Casa J. M. Garcia Feijoo J. Vidal-Sanz M. (2021b). Photosensitive ganglion cells: a diminutive, yet essential population. Arch. Soc. Esp. Oftalmol. 96, 299–315. doi: 10.1016/j.oftale.2020.06.020 34092284

[ref104] Vorwerk C. K. Kreutz M. R. Bockers T. M. Brosz M. Dreyer E. B. Sabel B. A. (1999). Susceptibility of retinal ganglion cells to excitotoxicity depends on soma size and retinal eccentricity. Curr. Eye Res. 19, 59–65. doi: 10.1076/ceyr.19.1.59.5336, PMID: 10415458

[ref105] Vorwerk C. K. Zurakowski D. McDermott L. M. Mawrin C. Dreyer E. B. (2004). Effects of axonal injury on ganglion cell survival and glutamate homeostasis. Brain Res. Bull. 62, 485–490. doi: 10.1016/S0361-9230(03)00075-3, PMID: 15036562

[ref106] Wang S. Gu D. Zhang P. Chen J. Li Y. Xiao H.. (2018). Melanopsin-expressing retinal ganglion cells are relatively resistant to excitotoxicity induced by N-methyl-d-aspartate. Neurosci. Lett. 662, 368–373. doi: 10.1016/j.neulet.2017.10.055, PMID: 29102785

[ref107] Whiteley S. J. Sauve Y. Aviles-Trigueros M. Vidal-Sanz M. Lund R. D. (1998). Extent and duration of recovered pupillary light reflex following retinal ganglion cell axon regeneration through peripheral nerve grafts directed to the pretectum in adult rats. Exp. Neurol. 154, 560–572. doi: 10.1006/exnr.1998.6959 9878191

[ref108] Zhao M. Toma K. Kinde B. Li L. Patel A. K. Wu K. Y.. (2023). Osteopontin drives retinal ganglion cell resiliency in glaucomatous optic neuropathy. Cell Rep. 42:113038. doi: 10.1016/j.celrep.2023.113038, PMID: 37624696 PMC10591811

